# Psychometric Properties of the Breast Cancer Awareness Measure (Breast-CAM): A Systematic Review and Meta-Analysis

**DOI:** 10.3390/cancers18060956

**Published:** 2026-03-15

**Authors:** Andrea Fejer, Mohammad Amin Atbaei, Afshin Zand, Timea Varjas, Zsuzsanna Kiss

**Affiliations:** 1 Department of Public Health Medicine, Medical School, University of Pecs, 7622 Pecs, Hungary; timea.varjas@aok.pte.hu (T.V.); zsuzsa.orsos@aok.pte.hu (Z.K.); 2 Department of Anesthesia, SEHA—Sheikh Khalifa Medical City, Al Karamah Street, Abu Dhabi P.O. Box 51900, United Arab Emirates; moamin@seha.ae

**Keywords:** breast cancer, breast cancer awareness, breast cancer awareness measure (Breast-CAM), early detection, systematic review, meta-analysis, psychometric properties

## Abstract

**Background/Objectives**: Breast cancer awareness is essential for early detection and timely help-seeking among women and represents a key component of multidisciplinary breast cancer prevention. The Breast Cancer Awareness Measure (Breast-CAM) is widely used to assess awareness of breast cancer symptoms, risk factors, and screening behaviors. Its measurement quality across populations has not yet been comprehensively evaluated. As Breast-CAM is a population-reported measurement instrument, evaluation using a standardized framework for measurement properties is required. This systematic review and meta-analysis aimed to assess the psychometric properties of the Breast-CAM across diverse populations and cultural adaptations, in accordance with COSMIN (COnsensus-based Standards for the selection of health Measurement INstruments) methodological standards. **Methods:** Major bibliographic databases and trial registries were systematically searched for peer-reviewed English-language studies published between 2010 and 2025 that evaluated at least one psychometric property of the Breast-CAM in adult women. Methodological quality was assessed using the COSMIN Risk of Bias checklist. Measurement properties were evaluated according to COSMIN criteria, and the certainty of evidence was graded using a modified GRADE approach. Meta-analysis was performed when data were sufficiently comparable. **Results:** Seventeen studies met the inclusion criteria for narrative synthesis, of which eleven were included in a meta-analysis, representing fourteen cultural adaptations of the instrument. A descriptive random-effects meta-analysis of reported Cronbach’s α yielded a pooled estimate of 0.89 (95% confidence interval 0.85–0.92). This value should be interpreted cautiously, as structural validity was frequently insufficient across cultural adaptations, limiting interpretation of internal consistency according to COSMIN guidance. Other measurement properties, including reliability and measurement error, were frequently inadequately assessed or unreported. The certainty of evidence ranged from very low to moderate. **Conclusions**: Content validity was generally rated as sufficient, although certainty of evidence was low. Despite the high pooled α estimate, the reliability of Breast-CAM cannot be firmly established because structural validity was frequently insufficient across cultural adaptations. In accordance with the COSMIN ceiling rule, internal consistency was not considered sufficient in the absence of adequate structural validity. Key measurement properties, including test–retest reliability, measurement error, and responsiveness, were rarely evaluated. Further high-quality psychometric studies, particularly in culturally diverse populations, are needed to address these gaps and support appropriate use of the instrument in research and public health practice.

## Simple Summary

Breast cancer is a major health concern for women worldwide, and early detection greatly improves survival. Awareness of breast cancer symptoms, risk factors, and screening helps women seek timely medical care. The Breast Cancer Awareness Measure is a commonly used questionnaire to assess how well women understand these important aspects of breast cancer. Despite its widespread use, the quality of evidence supporting this tool has not been comprehensively evaluated. This study aims to systematically review and combine existing research on how accurately and reliably the Breast Cancer Awareness Measure assesses breast cancer awareness among adult women. By summarizing and evaluating the available evidence, this research provides clear guidance on the strengths and limitations of this tool. The findings may support researchers, healthcare professionals, and policymakers in developing multidisciplinary breast cancer education, prevention, and early detection strategies.

## 1. Introduction

Breast cancer is the most common cancer among women worldwide, with ~2.3 million cases and ~670,000 deaths each year globally [1,2,3]. Rates are predicted to rise further to ~3 million cases and ~1 million deaths by 2040–2050 based on demographic projection models that account for population growth and aging [4]. Beyond its high incidence, breast cancer imposes a significant burden on global public health [3]. Despite substantial advances in breast cancer therapy, disparities in survival persist worldwide. These disparities are associated with differences in awareness, early detection, and access to timely diagnosis and treatment [5,6]. Breast cancer awareness and screening behaviors have been linked to earlier detection and earlier presentation [7]. Public knowledge of breast cancer warning signs, risk factors, and screening recommendations may contribute to effective prevention strategies, psychosocial support, and population-level cancer control.

Accurate measurement of awareness is essential for evaluating health promotion efforts, designing interventions, and informing policy. These aims require instruments that are not only psychometrically robust but also culturally appropriate. The Breast Cancer Awareness Measure (Breast-CAM), developed in 2009 by Cancer Research UK in collaboration with King’s College London and University College London [8], was created as a standardized tool to assess awareness of breast cancer symptoms, risk factors, and help-seeking behaviors. Since its introduction, Breast-CAM has been translated and adapted for use in multiple linguistic and cultural contexts. Yet, the quality and methodological consistency of these adaptations—particularly their reliability, structural validity, and responsiveness—have not been synthesized. Most existing evidence comes from single-country validation studies, limiting conclusions about the instrument’s consistency across settings. As a result, researchers and practitioners face uncertainty when selecting appropriate versions for use at local, regional, or international levels. To address this gap, this systematic review and meta-analysis aims to evaluate the psychometric properties of the Breast Cancer Awareness Measure (Breast-CAM) in adult women across diverse populations and settings. We specifically assessed reliability, validity, and responsiveness, and examined cultural adaptations for non-UK and multilingual populations. By synthesizing the psychometric evidence for the Breast Cancer Awareness Measure, this study supports multidisciplinary breast cancer research by informing prevention strategies, awareness-based interventions, and population-level early detection efforts.

## 2. Materials and Methods

### 2.1. Followed Guidelines

This systematic review was conducted and reported in accordance with the PRISMA 2020 statement for reporting systematic reviews [9] and the PRISMA-COSMIN for OMIs 2024 extension for systematic reviews of outcome measurement instruments [10]. PRISMA-COSMIN is a recently developed reporting guideline specifically intended for systematic reviews evaluating measurement properties of health-related instruments. The completed PRISMA-COSMIN checklist is provided as Supplementary Material (Supplementary File S1: PRISMA-COSMIN 2024 checklist for systematic reviews of outcome measurement instruments.) The database search strategy adhered to the PRISMA-S extension [11], which provides structured guidance for transparent reporting of search methods. Methodological appraisal and synthesis followed the COSMIN [12] framework, including use of the COSMIN Risk of Bias checklist [13] for assessing study quality, the COSMIN criteria for rating measurement properties, and the COSMIN-adapted GRADE approach to evaluating the certainty of evidence [12]. The study protocol was registered in PROSPERO (CRD420251158142).

### 2.2. Eligibility Criteria

Studies were eligible for inclusion if they met the following criteria. The target population consisted of adult women (≥18 years) from any country or setting. Eligible studies employed the Breast Cancer Awareness Measure (Breast-CAM), including the original instrument or any culturally adapted version. Quantitative study designs were included if they evaluated at least one psychometric property of the Breast-CAM or reported its cross-cultural adaptation. Psychometric properties of interest included reliability (internal consistency, test–retest or inter-rater reliability, and measurement error), validity (content, structural, construct, or criterion validity), and responsiveness, as defined by COSMIN guidelines. Studies published in English between January 2010 and October 2025 were considered eligible.

Studies were excluded if they involved children, adolescents, mixed-gender samples, or male or transgender participants. The Breast Cancer Awareness Measure was originally developed to assess breast cancer awareness among women. According to the instrument developers, a version designed to measure men’s awareness of breast cancer in women is currently under development [8]. The use of the Breast-CAM in populations other than women does not align with its intended and validated application. Publications were also excluded if they reported breast cancer awareness levels without assessing measurement properties or adaptation procedures, or if they were qualitative studies, narrative reviews, conference abstracts, or lacked an accessible full text. Studies not employing the Breast-CAM or using unrelated awareness measures were excluded. Non-English language publications were excluded to ensure accurate COSMIN-based assessment of psychometric properties, as some such studies lacked sufficient methodological detail and reliance on translated data could have affected interpretation. Multiple culturally adapted versions of the Breast-CAM from diverse linguistic and regional populations were included in this review. However, limiting the review to English-language publications may still have introduced language bias. [14] Validation studies published only in local languages may have been missed. Conclusions regarding global cross-cultural validity should be interpreted with caution. This decision was considered necessary to maintain methodological rigor. Studies that did not report sufficient psychometric or adaptation data to allow extraction or COSMIN evaluation were excluded.

### 2.3. Information Sources/Electronic Databases

A comprehensive search was conducted across multiple electronic databases and trial registries to identify studies evaluating the psychometric properties of the Breast-CAM. The following databases were systematically searched between 15 September and 15 October 2025, with coverage from January 2010 to 15 October 2025: PubMed/MEDLINE, Scopus, the Web of Science Core Collection, Embase, CINAHL (via EBSCOhost), the Cochrane Library, and Google Scholar. Trial and study registries: ClinicalTrials.gov, the WHO International Clinical Trials Registry Platform (ICTRP), the ISRCTN Registry, and the EU Clinical Trials Register (EU-CTR). The final database and registry searches were completed on 15 October 2025 (Table 1).

### 2.4. Additional Sources

Preprints identified during the search were screened but were not eligible for inclusion because they did not report psychometric properties of the Breast-CAM. All information sources and search dates were documented in line with PRISMA-S [11] and PRISMA-COSMIN OMI 2024 guidelines [10].

### 2.5. Search Strategy

We searched using customized strategies for each source. The following section outlines the exact terms, filters, and limits used to capture all relevant Breast-CAM studies. The search strategy was developed and independently reviewed by two members of the research team to identify empirical studies evaluating the psychometric properties of the Breast Cancer Awareness Measure (Breast-CAM). Elements of the PICO framework were adapted to structure the search strategy, focusing on the target population, construct, and outcome measurement instrument: (“breast cancer”/exp OR “female”/exp) AND (“bcam” OR “breast cancer awareness measure” OR “breast cam” OR “validated questionnaire”/mj OR “questionnaire”/exp OR “tool”/exp) AND (“breast cancer awareness” OR “breast cancer knowledge”) AND [2010–2025]/py. Reference lists of included studies and citation tracking were screened to ensure that key relevant studies were captured.

### 2.6. Database Search Overview

Searches were performed in PubMed/MEDLINE, Scopus, Web of Science, Embase, CINAHL (via EBSCOhost), the Cochrane Library, and Google Scholar (screening the first 200 results ranked by relevance) using customized strategies for each platform, accounting for differences in indexing and syntax. This limit was applied because Google Scholar ranks results by relevance, and relevant studies are typically concentrated within the initial results. Comprehensive searches were already conducted in multiple bibliographic databases to ensure broad coverage. Each database was searched for peer-reviewed studies published in English between January 2010 and 15th of October 2025, focusing on adult women. Full search strings and database-specific adaptations are provided in Supplementary File S2. Search strategy to ensure transparency and reproducibility.

### 2.7. Selection Process

All search results were first imported into Zotero, where duplicate records were identified and removed. De-duplicated records were then exported to ASReview, (Active Learning for Systematic Reviews) [15] as a screening support tool during the title and abstract screening phase. ASReview was applied after duplicate removal to assist prioritization of records based on relevance predictions. An initial training set was created by manually labeling a subset of records as relevant or irrelevant based on the predefined eligibility criteria. The model was updated as screening progressed, using additional records screened and labeled by the reviewers, allowing gradual refinement of relevance predictions. Screening was conducted using the default machine-learning model with human-in-the-loop active learning, and all inclusion and exclusion decisions were made by reviewers, not by software. In total, 1229 records (after duplicate removal) were screened with the support of ASReview, as shown in the PRISMA flow diagram. The tool was used only to prioritize the order of review, while all inclusion and exclusion decisions were made by the reviewers. Screening continued until no further potentially relevant records were identified, and included studies were checked against the screened set to ensure that relevant studies were not missed.

Screening was conducted in two stages:

1. Title and abstract screening:

Two reviewers (from a team of five) independently screened titles and abstracts within ASReview. The tool was used only to prioritize records; all inclusion and exclusion decisions were made by the reviewers based on the predefined eligibility criteria. Disagreements were resolved through discussion.

2. Full-text screening:

The same two reviewers independently assessed full-text articles for eligibility. A third reviewer was available to adjudicate unresolved disagreements, although consensus was achieved in most cases.

The eligibility criteria described above were applied consistently. ASReview was used solely to support efficiency and did not replace independent reviewer judgment or predefined eligibility criteria.

#### 2.7.1. Data Collection Process

Data extraction was carried out using a structured form developed in line with COSMIN recommendations [12]. The form was piloted on four representative studies (two describing development or cultural adaptation of the Breast-CAM and two evaluating its measurement properties) to ensure that all relevant domains were captured; minor revisions were made for clarity.

Two reviewers independently extracted data from each included study, recording:

- Study design and setting;
- Population characteristics;
- Version and language of the Breast-CAM;
- Measurement properties assessed;
- Statistical methods used and numerical psychometric outcomes.

Extraction followed PRISMA-COSMIN OMI reporting guidance [10]. Discrepancies were resolved by discussion, with a third reviewer available if needed. Formal inter-rater agreement statistics (e.g., Cohen’s kappa) were not calculated. No automation or AI tools were used for data extraction. All data was managed in Microsoft Excel.

#### 2.7.2. Data Items

From each included study, we extracted:

- Study characteristics: First author, year, country, design (development, translation, validation), and sample size.
- Population characteristics: Key demographics (e.g., age range, recruitment setting) and inclusion criteria where available.
- Instrument details: Breast-CAM version, language, and any reported adaptation procedures.
- Measurement properties and results: Internal consistency, test–retest reliability, construct validity, responsiveness, statistical methods, and main findings (α, ICC, factor loadings, hypothesis-testing results).

### 2.8. Study Risk of Bias Assessment

The methodological quality of the included studies was assessed using the COSMIN Risk of Bias checklist [13] for studies on measurement properties, in line with PRISMA-COSMIN OMI 2024 guidance [10].

Two reviewers independently evaluated each study across all relevant measurement properties (e.g., content validity, structural validity, internal consistency, reliability, measurement error, hypothesis testing, responsiveness, cross-cultural validity). Each property was rated using the COSMIN four-point scale (very good, adequate, doubtful, inadequate), applying the “worst score counts” principle [13]. When a study reported several measurement properties with different quality ratings, each property was assessed separately according to COSMIN criteria. Differences between reviewers were resolved through discussion, with a third reviewer consulted if needed. The final rating for each property was based on the reviewers’ agreement.

All ratings were recorded in a structured Excel sheet (Supplementary File S3—COSMIN Risk of Bias and Measurement Properties Extraction Sheet), which included study characteristics, Breast-CAM version, psychometric properties evaluated, COSMIN ratings per property, and an overall risk of bias judgment. No automation or AI was used in this step.

#### Measurement Properties

In line with COSMIN methodology [12,16], content validity reflects the degree to which items adequately represent the breast cancer awareness construct for the target population. Structural validity assesses whether the dimensional structure of the instrument corresponds to the construct being measured. Internal consistency evaluates the interrelatedness of items within a unidimensional scale, while reliability reflects score stability in stable individuals over time. Measurement error quantifies random and systematic error not attributable to true change. Construct and criterion validity assess whether scores behave as theoretically expected in relation to other variables or a gold standard, respectively. Responsiveness evaluates the ability of the instrument to detect meaningful change over time, and cross-cultural adaptation examines equivalence across language and cultural versions [12].

Each measurement property of the Breast-CAM was evaluated in accordance with COSMIN standards for outcome measurement instruments [12]. For each included study, we examined evidence for internal consistency, reliability, structural validity, content validity, and hypothesis-testing construct validity. Measurement results were judged using the COSMIN criteria for good measurement properties, applying thresholds such as Cronbach’s α or intraclass correlation coefficients (ICC) ≥ 0.70 [12], appropriate model-fit indices for CFA or IRT models, and adequate factor loadings for structural validity. While α values ≥ 0.70 were considered acceptable, values exceeding 0.95 were interpreted with caution, as very high internal consistency may suggest item redundancy. Based on these criteria, each finding was classified as sufficient (+), insufficient (−), or indeterminate (?), following COSMIN decision rules [12,17].

Two reviewers independently assigned ratings, resolving disagreements through discussion; a third reviewer was available when consensus could not be reached. Ratings were synthesized by measurement property to provide an overall picture of the evidence base. Where quantitative pooling was not feasible due to methodological heterogeneity or incomplete reporting, results were narratively synthesized instead. The certainty of evidence for each measurement property was subsequently evaluated using the COSMIN-adapted GRADE approach, considering risk of bias, inconsistency, imprecision, and indirectness. All evaluations and grading decisions were performed manually to ensure transparent, context-appropriate interpretation. The Summary of Measurement Properties (SOMP) and COSMIN Rating Criteria are presented in Table 2.

### 2.9. Synthesis Methods

After full-text inclusion, studies were grouped according to the measurement properties they reported (e.g., internal consistency, structural validity, reliability, content validity). Only studies providing empirical data suitable for COSMIN-based rating were included in the synthesis for that property.

Studies that evaluated multiple properties contributed to each relevant synthesis. Where studies described properties qualitatively (e.g., “reliable” or “valid” without numerical data), they were included in narrative summaries but excluded from quantitative pooling. This approach followed COSMIN recommendations to ensure that each synthesis was based on analyzable and methodologically acceptable data [12].

For measurement properties with sufficient homogeneous quantitative data, we conducted a random-effects meta-analysis using the metafor package (version 4.8.0) in R (version 4.5.1; R Foundation for Statistical Computing, Vienna, Austria). [18]. For internal consistency, Cronbach’s α coefficients were pooled using appropriate variance estimation methods (e.g., Bonett’s approach), and 95% confidence intervals were obtained from random-effects models [19,20]. Cronbach’s α is influenced by the number of items and the underlying factor structure. Accordingly, values from different Breast-CAM versions may not be directly comparable and were interpreted cautiously.

Statistical heterogeneity was examined using Q, τ^2^, and I^2^. When pooling was not feasible due to heterogeneity in methods, populations, or Breast-CAM versions, we used narrative synthesis. In these cases, we summarized: (a) direction and magnitude of findings, (b) COSMIN Risk of Bias ratings, and (c) sufficiency ratings (+/−/?). Certainty of evidence for each measurement property was assessed using the COSMIN-modified GRADE approach [12].

Where sufficient studies were available, we explored potential sources of heterogeneity qualitatively, considering differences in language versions, sample characteristics, and study design. Because of the limited number of studies per subgroup, formal subgroup meta-analysis was not performed.

To assess robustness, we conducted leave-one-out sensitivity analyses for the internal consistency meta-analysis, re-estimating pooled Cronbach’s α after sequentially removing each study. This tested whether any single study had a disproportionate influence on summary estimates.

### 2.10. Certainty Assessment

We assessed the certainty of the evidence for each measurement property of the Breast-CAM using the COSMIN-modified GRADE approach [12]. This method evaluates four domains: risk of bias, inconsistency, imprecision, and indirectness. Risk of bias was judged using the COSMIN Risk of Bias checklist [13], focusing on the appropriateness of psychometric methods, sample adequacy, and adherence to recommended validation procedures. Inconsistency was assessed by comparing the direction and magnitude of results across cultural versions and analytical approaches. Imprecision was evaluated according to total sample size and the width of confidence intervals, with higher certainty assigned to results supported by larger and more precise estimates. Indirectness was considered when study populations or validation contexts differed from the intended application of the Breast-CAM as an awareness tool.

Certainty ratings were assigned at the level of each measurement property, not at the study level, and were expressed as high, moderate, low, or very low. Following COSMIN guidance, the ceiling rule was applied such that the certainty of evidence for internal consistency did not exceed that of structural validity. This reflects the principle that reliability is interpretable only when dimensionality is adequately supported, regardless of the magnitude of pooled Cronbach’s α values [12]. Evidence was downgraded when one or more domains displayed serious limitations. Two reviewers performed the GRADE assessments independently; disagreements were resolved by discussion, with a third reviewer available when necessary. No deviations from the COSMIN framework were introduced. Table 3 presents the COSMIN-modified GRADE decision rules used to rate the certainty of evidence for each measurement property.

### 2.11. Formulating Recommendations

Recommendations on the use of the Breast Cancer Awareness Measure (Breast-CAM) were developed using a predefined framework. This framework linked the certainty of evidence for each measurement property to its intended use. Certainty was assessed using the COSMIN-modified GRADE approach [12].

Following COSMIN guidance [12], measurement properties with high, moderate, or low certainty evidence were considered suitable for population-level applications. These included awareness monitoring and evaluation of public health interventions. Measurement properties supported by very low certainty evidence were not considered suitable for individual-level use. This included clinical decision-making and the assessment of change over time. A summary of recommended applications of the Breast-CAM based on certainty of evidence is presented in Table 4.

Final recommendations were based on three factors: the certainty of evidence, the specific measurement property, and the intended context of use. This approach ensured consistency with COSMIN standards for outcome measurement instruments.

## 3. Results

### 3.1. Study Selection

A total of 1446 records were identified across all database searches and supplementary sources. After removing duplicates, 1229 records were screened by title and abstract. A total of 115 full-text articles were assessed for eligibility using predefined COSMIN criteria [12].

**Figure 1:**
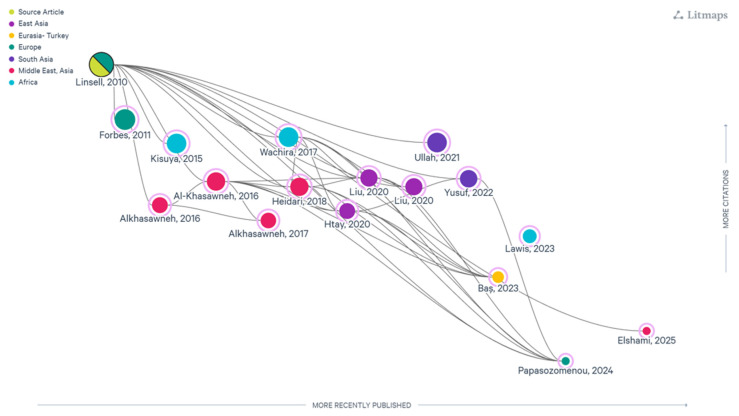
LitMaps. Visualization of included studies related to the Breast-CAM. Nodes represent studies and connections indicate citation relationships between studies [8,21,22,23,24,25,26,27,28,29,30,31,32,33,34,35,36].

Following full-text screening, 17 study reports met the inclusion criteria, each providing empirical evidence on at least one psychometric property of the Breast Cancer Awareness Measure (Breast-CAM). These studies represented a range of cultural and linguistic adaptations, including versions from the UK, Oman, Kenya, Iran, China, Malaysia, Pakistan, Turkey, Greece, Brunei, Sudan, and Palestine. A LitMaps visualization illustrating conceptual linkages across the 17 included studies (e.g., cultural adaptation methods, measurement properties assessed, and study designs) is provided in Figure 1 to support visualization of the evidence landscape.

Of the 17 included studies, 11 reported Cronbach’s α estimates that were sufficiently comparable to allow a descriptive random-effects meta-analysis of reported α values. Estimates were considered comparable when they referred to the total scale or clearly defined subscales of the Breast-CAM and were calculated in adult female populations. They also needed to include sufficient statistical information (e.g., sample size) to permit variance estimation. The remaining studies contributed to the narrative syntheses of other measurement properties such as structural validity, content validity, reliability, construct validity, responsiveness, and measurement error.

All included study reports evaluated the Breast-CAM or one of its culturally adapted versions. No alternative OMIs met the inclusion criteria for this review, which focused on the Breast-CAM and its adaptations.

**Figure 2:**
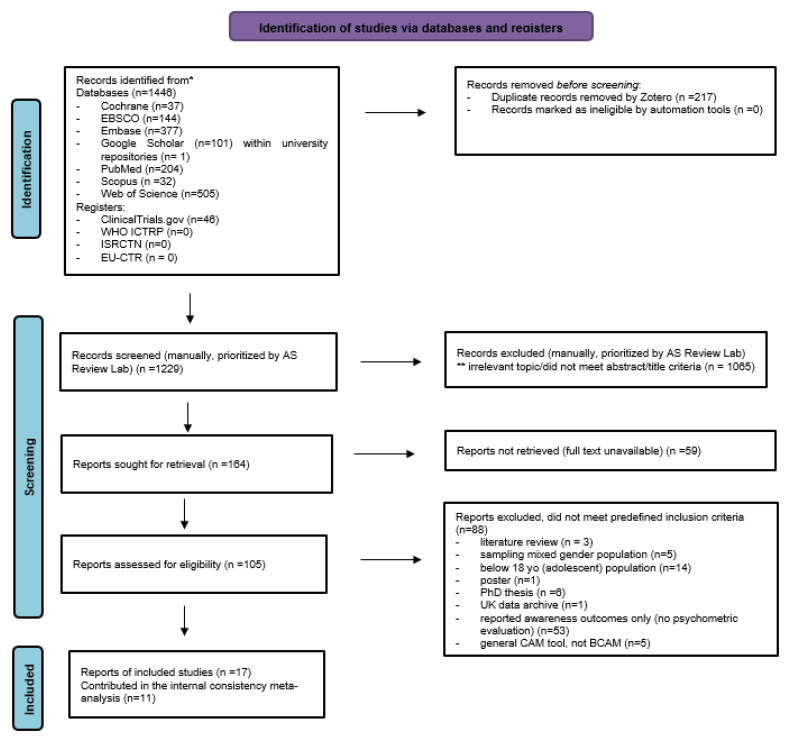
PRISMA FLOW DIAGRAM.

A PRISMA flow diagram [9] summarizing the search, screening, and inclusion process is presented in Figure 2.

A total of 88 full-text articles were excluded for predefined reasons. A complete list of excluded articles and reasons for exclusion is provided in Supplementary Material (Supplementary Appendix S1).

### 3.2. OMI Characteristics

The included studies used multiple culturally adapted versions of the Breast Cancer Awareness Measure (Breast-CAM), all derived from the original UK instrument. Versions were translated and adapted to local languages and contexts and were administered as self-report questionnaires in community or healthcare settings. Wording, format, and item numbers varied slightly across versions. The core domains were similar and generally covered symptom recognition, risk factors, screening awareness, and barriers to help-seeking.

Many adaptations used recognized translation and cultural-adaptation procedures, such as forward–backward translation, expert review, and pilot testing [37]. In some cases, documentation was incomplete and these procedures were not always described in detail.

Samples in the development or validation studies generally included adult women recruited from community, clinical or screening settings. These instruments were designed for population-level assessment. They are useful for describing and comparing patterns of breast cancer awareness across different domains, but not for precise interpretation of individual scores. This is consistent with COSMIN guidance on reliability and interpretability requirements and established measurement principles [12,38].

Key characteristics of each version, including settings, translation procedures, and assessed psychometric properties, are summarized in Table 5.

### 3.3. Interpretability Aspects for Each Included OMI

Across the 17 included studies, interpretability information for the various BCAM versions was limited and often inconsistently reported. Most studies described item-level percentages of correct recognition for symptoms, risk factors, or screening behaviors rather than providing summary scores, measures of score distribution, or thresholds for meaningful improvement [13]. Specifically, no studies established minimal important change (MIC) or minimal important difference (MID) values, preventing interpretation of whether observed score differences represent meaningful change. Only a few adaptations, such as the Persian and Chinese versions, included numeric score distributions (e.g., means, medians, IQRs) that allow a clearer understanding of where respondents typically scored.

Missing data reporting was generally poor. Most authors mentioned high completion rates, but only a minority provided exact percentages. Formal floor or ceiling analyses were rare. In several studies, classical symptoms showed high recognition (ceiling-like patterns), while age-related risk or less common signs showed very low recognition (floor-like patterns). Consequently, the ≥15% threshold recommended by COSMIN could not be consistently applied because sufficient quantitative data were usually not reported [12,38].

Change-score interpretability was available only for intervention studies, which reported improvements after educational programs or mobile-app exposure. None of the included studies established a minimally important change (MIC) or minimally important difference (MID) [12]. No study attempted to define patient-important thresholds, responder definitions, or cut-points for interpretation of total or domain score.

Interpretability evidence across settings remains limited and inconsistent, with most Breast-CAM versions reporting only descriptive percentages rather than the structured interpretability metrics recommended by COSMIN [12]. Future validation studies should determine minimal important change (MIC) or minimal important difference (MID) values to allow meaningful interpretation of score levels and changes over time. Interpretability results for each Breast-CAM version are presented in Table 6.

### 3.4. Feasibility Aspects for Each Included OMI

Across the 17 studies, all BCAM versions were feasible to use in both research and community settings, consistent with feasibility considerations recommended for outcome measurement instruments [38,39]. Most instruments were delivered as paper questionnaires, either self-completed or interviewer-administered when literacy was limited. This flexibility made the measures usable across a wide range of populations, including older adults in the UK, women with low literacy in Kenya and Oman, and rural groups in Sudan and Palestine. Completion was consistently high, and none of the studies reported major difficulties in understanding or responding to items.

The length of the instruments varied only slightly. Full BCAM versions included symptoms, risk-factor, age-risk and help-seeking blocks, while several adaptations used shorter modules tailored to local education or intervention programs. Despite these differences, all versions remained manageable and were considered appropriate for routine data collection.

Response formats were simple. Nearly all questionnaires relied on *yes/no/don’t know* or brief multiple-choice options, with occasional Likert-type questions. No study reported the need for special mental or physical abilities beyond basic comprehension or the ability to respond orally during interviews. Interviewer administration was especially valuable in settings where literacy was variable.

Scoring procedures were straightforward. Many studies used summed scores or simple counts of correct responses, while others relied only on item-level percentages. Psychometric studies that used derived domain scores or 0–100 transformations described these procedures clearly, and none required specialized software for routine scoring.

All adaptations acknowledged Cancer Research UK as the original source of the BCAM. No copyright restrictions affected feasibility.

The BCAM and its international adaptations were practical, acceptable and easy to administer across diverse populations and study designs. Feasibility results for each Breast-CAM version are presented in Table 7.

### 3.5. Study Characteristics

Seventeen studies met the inclusion criteria and reported at least one measurement property of BCAM or its adapted versions. The original BCAM was developed in the United Kingdom to assess women’s awareness of breast cancer symptoms [8]. It was later used without modification in an intervention trial with older women [22].

Several studies focused on adapting BCAM to local cultural and linguistic contexts. A full Arabic adaptation was completed in Oman [24], using forward–back translation, expert review, and pilot testing. The validated Arabic version was then used in community samples [23] and applied in tailored health education programs [25]. Comparable translation and validation processes were carried out for Swahili in Kenya [26], Persian in Iran [27], Mandarin Chinese in China [28], Malay in Malaysia [30] and Turkish in Turkey [33].

Other studies used BCAM as an outcome tool rather than re-evaluating its psychometric performance [22,29,32]. A smaller group of studies introduced localized modifications to fit regional contexts, such as the Sudanese adaptation [34], a Greek version developed for rural communities [35], and a modified Arabic version used in a nationwide Palestinian survey [36].

Across all studies, most instruments were administered to adult women in community, clinic, screening, or digital health settings. Only a subset reported quantitative measurement properties suitable for meta-analysis (e.g., Cronbach’s α with sufficient structural validity). The full characteristics of each included study, including adaptation method, sample details, and psychometric evidence, are summarized in Table 8.

### 3.6. Assessment of Risk of Bias in Studies

Risk of bias assessments showed considerable variation across the included studies. Instruments that performed a full psychometric evaluation generally demonstrated stronger methodological quality. The Persian BCAM [27] and the Turkish BCAM-Tr [33] received very good ratings across multiple domains, reflecting the use of established structural validity procedures, high internal consistency within confirmed factor structures, and appropriate test–retest reliability. The Chinese C-BCAM [28] validation article also showed a solid methodological foundation, although gaps remained in responsiveness and measurement error.

A second group of studies focused primarily on translating and adapting the Breast-CAM for local use, with more limited evaluation of its measurement properties. These studies—including the Arabic adaptations [23,24,25] and the Swahili BCAM validation [26]—demonstrated that the instrument was culturally and linguistically appropriate and provided evidence for internal consistency and, in some cases, structural validity. The key properties such as measurement error and responsiveness were rarely examined, and reliability testing was applied inconsistently across studies. As a result, methodological quality ratings for these studies generally fell within the adequate to doubtful range.

The weakest evidence emerged in studies that modified BCAM items or used translated versions without re-establishing psychometric characteristics. Modified Arabic [34] and Greek [35] versions, for example, were implemented after small pilots or expert opinion but lacked structural validation and repeated measures, resulting in predominantly inadequate ratings. Across the dataset, measurement error and responsiveness were the least frequently reported domains, and reliability was inconsistently assessed outside comprehensive validation studies. Hypothesis testing and cross-cultural adaptation showed the most variability, with higher ratings when predefined expectations were evaluated rather than exploratory associations.

Methodological quality tended to align with study purpose: validation studies were stronger, while applications of BCAM as an outcome tool or context-specific modifications without re-assessment showed greater risk of bias. The COSMIN risk-of-bias ratings for studies that reported evaluable measurement properties are shown in Table 9.

### 3.7. Results of Individual Studies

Structural validity evidence was mainly available from the subset of studies that conducted factor analysis and reported fit indices. The Persian BCAM [27] provided the most comprehensive evidence, combining exploratory and confirmatory factor analyses with excellent model fit (e.g., RMSEA = 0.046, CFI = 0.984). The Chinese [28] and Turkish [33] versions also demonstrated acceptable factor structures and fit statistics, supporting dimensional stability. Versions that were translated and implemented without testing latent structure—such as the Omani [23,24,25], Sudanese [34], and Greek [35] adaptations—were rated insufficient for structural validity regardless of their reliability values. This follows the COSMIN rule that internal consistency cannot be judged without evidence that items form a unidimensional factor.

Internal consistency was the most consistently supported measurement property across the included BCAM adaptations. Most instruments demonstrated alpha values above the COSMIN threshold of 0.70, which indicates generally high reported internal consistency estimates within individual versions. The Persian [27], Turkish [33], Malay [30], and Chinese BCAM [28,29] versions stood out, with Cronbach’s α values typically ranging from 0.84 to 0.94, indicating strong internal coherence across items and subscales. The Swahili BCAM [26] was the exception: α values differed meaningfully between domains. Knowledge items clustered well (α ≈ 0.80), but the “external barriers” domain produced a lower coefficient (α ≈ 0.60). Because COSMIN recommends domain-specific assessment when structural assumptions are unclear, this pattern was rated as indeterminate rather than insufficient.

Reliability evidence, although less frequently reported than internal consistency, supported acceptable stability in several versions. The Persian [27] and Turkish [33] BCAMs demonstrated strong test–retest reliability (ICC ≈ 0.84–0.89), and the Arabic-BCAM-A validation study [25] demonstrated high inter-rater reliability (r = 0.97). Other adaptations did not include repeated-measures testing, resulting in indeterminate ratings for this property.

Measurement error was directly quantified in one instrument (Persian BCAM [27]), where SEM and SDC were reported, however, MIC was not established. Measurement error remained indeterminate under COSMIN.

Construct validity varied widely. Several studies relied on demographic regressions or post hoc associations (e.g., age, education, income), which do not meet COSMIN criteria because they do not evaluate predefined hypotheses about the direction or magnitude of relationships. Such exploratory approaches were rated as indeterminate. Only versions that explicitly compared theoretically distinct groups—such as the Turkish [33] known-groups analysis—or evaluated discriminant and convergent validity, as in the Persian BCAM [18], were rated sufficient.

Criterion validity, evidence was limited and based on indirect comparators. The Persian BCAM [27] demonstrated strong ability to distinguish between medical experts and the general population (AUC = 0.822), and the original Arabic adaptation [24] showed meaningful correlations with established indicators.

Responsiveness was limited; only the Malay BCAM used in a mobile-app intervention [32] demonstrated pre–post changes.

Cross-cultural adaptation procedures were generally well executed across the translated versions. Most studies applied internationally accepted translation frameworks—such as forward–back translation, expert panel review, and cognitive interviewing—as seen in the Arabic, Persian, Chinese, Malay, Turkish, and Greek adaptations. These procedures generally fulfilled COSMIN criteria for cultural equivalence. Adaptations relying mainly on expert review, showed lower methodological robustness, resulting in adequate to very good ratings. A few studies relied on expert review alone (e.g., Sudanese adaptation), which led to lower ratings due to limited pilot testing or lack of cognitive debriefing.

The reported results for each measurement property, as assessed in the included studies, are summarized in Table 10.

### 3.8. Results of Synthesis

#### 3.8.1. Reliability (Internal Consistency, Test–Retest Reliability, Measurement Error)

**Figure 3:**
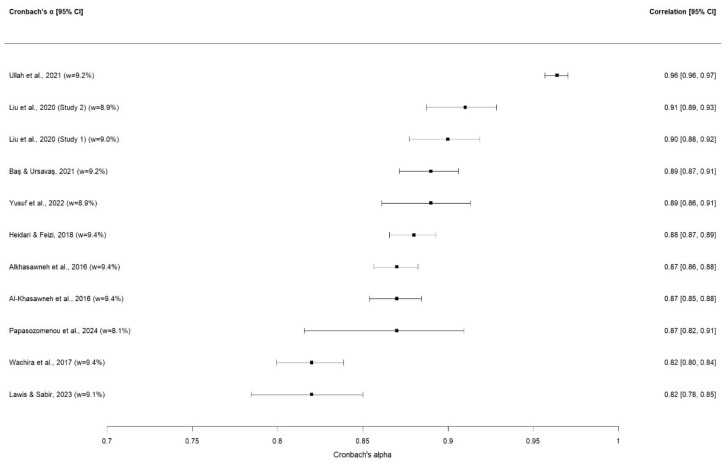
Forest plot of Cronbach’s α coefficients with 95% confidence intervals for 11 included Breast-CAM studies [23,24,26,27,28,29,31,32,33,34,35].

**Figure 4:**
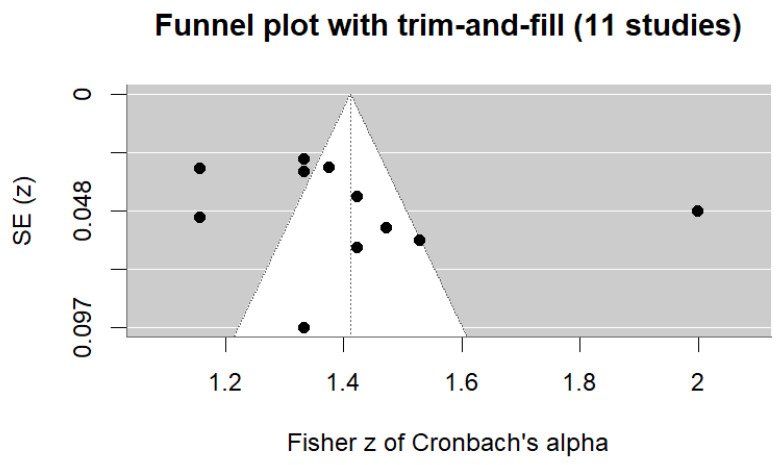
Funnel plot of Fisher’s z-transformed Cronbach’s α values with trim-and-fill estimation. Black dots represent individual study estimates; the white triangular region represents the expected funnel around the pooled effect estimate and the grey shaded areas indicate regions outside the expected funnel.

A random-effects meta-analysis was performed on 11 Breast-CAM studies that reported Cronbach’s α estimates and provided sufficiently comparable data for quantitative synthesis. Where a single publication reported more than one independent internal consistency estimate, each estimate was entered separately in the meta-analysis. The pooled Cronbach’s α was 0.89 (95% CI 0.85–0.92). In line with COSMIN guidance, this pooled estimate was not interpreted as evidence of sufficient internal consistency because structural validity was frequently insufficient or inconsistently assessed across studies. Differences in item composition, adaptation procedures, and study populations further contributed to heterogeneity [40] (Figure 3). Individual α values ranged from 0.82 to 0.96, indicating generally high reported internal consistency estimates across studies. Substantial heterogeneity was observed (I^2^ = 97.2%; Q = 250.2, *p* < 0.001; τ^2^ = 0.055), indicating very high between-study variability. This suggests that differences in cultural adaptations, item composition, and study populations contributed meaningfully to variation in the reported α estimates. The 95% prediction interval (0.74–0.95) reflects variability in reported α values across studies. These values should not be interpreted as confirmation of adequate internal consistency in the absence of confirmed structural validity. Funnel plot inspection and trim-and-fill analysis showed no evidence of small-study effects. Interpretation is limited by the small number of studies and substantial heterogeneity. The pooled estimate remained unchanged (Figure 4). In line with COSMIN guidance, internal consistency was rated as indeterminate (?), because structural validity was insufficient or inconsistently assessed across versions. Applying the COSMIN ceiling rule, the certainty of evidence for internal consistency did not exceed that for structural validity and was judged as low. The pooled estimate represents a descriptive summary of reported α values rather than definitive evidence of sufficient internal consistency across Breast-CAM versions.

Each point represents the study-specific α estimate with its 95% confidence interval, scaled by inverse-variance weight. Multiple independent estimates from a single study are shown separately. The pooled random-effects estimate (α = 0.89, 95% CI: 0.85–0.92) is indicated on the summary line.

The results of the meta-analysis are summarized in Table 11 which presents the pooled effect size, heterogeneity indices, COSMIN overall rating, and GRADE certainty assessment.

At the study level, most Breast-CAM adaptations reported α ≥ 0.70 at scale or subscale level, including the Persian [27], Turkish [33], Malay [32], Chinese [28,29] and several Arabic versions [23,24,34] which often showed coefficients between 0.84 and 0.94. An exception was the Swahili [26] “external barriers” subscale, where α was around 0.60, while symptom domains remained acceptable. In versions where structural validity was not assessed, COSMIN recommends treating internal consistency findings as indeterminate rather than definitively sufficient, which is reflected in the summary ratings.

Evidence for test–retest reliability was more limited. Only a small number of versions—including the Persian [27], Turkish [33], and Chinese [28] Breast-CAM—reported test–retest metrics, typically with ICC or correlation coefficients ≥ 0.70 over appropriate intervals [41]. Sample sizes for retest subsamples were modest, and not all studies clearly prespecified intervals or conditions of administration. Other versions reported inter-rater correlations or cross-group comparisons under the label of “reliability,” which does not meet COSMIN criteria for test–retest reliability. When synthesized, reliability beyond internal consistency was rated as insufficient or indeterminate, and the certainty of evidence was judged very low, mainly due to imprecision, small sample sizes, and methodological limitations.

Measurement error was directly quantified in only one instrument (the Persian Breast-CAM [27]), where SEM and SDC statistics were reported but no minimal important change (MIC) was defined [38]. Without MIC, COSMIN recommends classifying measurement error as indeterminate. For all other versions, measurement error was either not estimated or only partially addressed through floor and ceiling effects. As a result, measurement error was rated indeterminate (?) overall, with very low certainty of evidence.

#### 3.8.2. Validity (Content Validity, Structural Validity, Construct Validity, Criterion Validity)

Across versions, content validity was generally supported but unevenly reported. Most adaptations employed formal translation and cultural adaptation procedures, such as forward–backward translation, expert panel review, and small-scale pilot or cognitive testing. Some versions (e.g., Turkish [33], Chinese [28], Persian [27]) additionally reported item- and scale-level content validity indices (I-CVI, S-CVI [42] with values in the acceptable or excellent range. Other adaptations described the process more briefly, without quantitative indices or user-testing details. When synthesized, content validity for the Breast-CAM was judged sufficient (+) at the overall instrument level. However, the certainty of evidence was low, reflecting indirectness due to limited involvement of target users in some settings and incomplete reporting across several studies.

Structural validity was systematically evaluated in only a subset of Breast-CAM versions. The Persian [27] and Chinese [28] instruments provided the most rigorous evidence, combining exploratory and confirmatory factor analyses with acceptable fit indices (e.g., RMSEA ≤ 0.06; CFI ≈ 0.94–0.98) [43] and coherent factor structures for warning signs, risk factors, and barriers. The Swahili BCAM [26] supported a plausible factor solution through EFA, and the Turkish version [33] reported CFA results for an 11-item one-factor model with fit indices around conventional thresholds. Most other adaptations, including Arabic [23,24], Pakistani [31], Sudanese [34], and Greek versions [35], did not evaluate latent structure beyond face or content evaluation. When considered together, these findings led to an overall COSMIN rating of insufficient (−) for structural validity, with low certainty, driven by the small number of high-quality factor-analytic studies and the absence of dimensional testing in many versions.

Findings for construct validity (hypothesis testing) were mixed. A few versions of the Breast-CAM, such as the Persian [27] and Turkish [33] adaptations, evaluated predefined hypotheses related to discriminant or known-groups validity [44]. These studies compared experts with lay women or health professionals with the general population and generally confirmed the expected differences or associations. Many studies relied on post hoc associations with demographic variables (e.g., age, education, income, previous contact with breast cancer) without formally stating a priori hypotheses or expected effect sizes. Under COSMIN criteria, such exploratory analyses cannot be rated as clear evidence of construct validity. Construct validity was synthesized as inconsistent or indeterminate (±/?), with low certainty of evidence due to inconsistent methods and a lack of prespecified hypotheses in most studies.

Criterion validity was rarely examined, and no true gold standard for breast cancer awareness exists. The Persian Breast-CAM [27] reported an AUC of 0.822 [45] in discriminating experts from general women, and one Arabic version [24] showed moderate correlations with an external criterion (r ≈ 0.58). The comparators used were not universally established standards, and such analyses were not replicated across settings. As a result, criterion validity was rated as insufficient (−) or indeterminate (?), with very low certainty. This limits confidence in the Breast-CAM’s performance when compared with external benchmarks.

Cross-cultural adaptation [46] processes, while not graded as a measurement property, were generally adequate in most versions and form an important foundation for interpreting the validity evidence. The uneven application of cognitive interviewing, user involvement, and pilot testing across settings contributed to the downgrading of certainty for several validity domains.

#### 3.8.3. Evidence on Responsiveness

Evidence for responsiveness of the Breast-CAM was limited to a small number of studies. The original UK intervention [21] format and the Malay app- or intervention-based versions [32] reported pre–post increases in awareness scores following educational programs, with changes in the expected direction. These results suggest that the instrument can detect change at the group level in the context of structured awareness interventions [12]. Designs were typically single-group pre–post or involved limited follow-up periods, and no study established MIC values or systematically linked change scores to meaningful behavioral outcomes (e.g., help-seeking, screening uptake) which limits interpretability of responsiveness under COSMIN standards [12,38].

When synthesized, responsiveness was therefore rated sufficient (+) in specific intervention contexts but supported by low certainty of evidence overall, due to imprecision, indirectness, and limited design diversity. The Breast-CAM appears suitable for evaluating changes in awareness in research or program settings, but the responsiveness evidence is not yet strong enough to guide individual-level interpretation of change.

### 3.9. Study-Level Evidence Synthesis

The detailed study-level ratings for each measurement property are presented in Supplementary Table S1, which summarizes the psychometric properties of individual BCAM versions in accordance with COSMIN recommendations. For each version, we report only those measurement properties that were empirically assessed, without imputing missing domains. For multidimensional instruments (e.g., Persian [27] and Chinese versions [28,29]), results are presented at the subscale level to ensure conceptual clarity and appropriate interpretation. Studies are organized chronologically (2016–2024) to illustrate the evolution of BCAM adaptation, structural models, and methodological rigor across cultural contexts. In versions where specific measurement properties were not evaluated, the table uses “–” to distinguish missing evidence from insufficient findings. COSMIN overall ratings (+/–/±/?) and PROM-adapted GRADE levels reflect synthesis per measurement property, preserving transparency in the evaluation of each domain [10,12,13].

### 3.10. Result of Inconsistency

No formal subgroup or meta-regression analyses were carried out. Although studies reported factors such as age, education, and setting, these characteristics were closely tied to specific Breast-CAM versions, making statistical comparisons inappropriate. Instead, we examined heterogeneity descriptively [47]. Versions that included proper structural validity testing (e.g., the Persian [27], Chinese [29], and Turkish [33] adaptations) showed more stable reliability estimates, while versions without factor analysis produced more variable results. Instruments adapted through rigorous translation and expert review also performed more consistently than minimally modified translations. Some variation was linked to sample types—large community samples tended to yield more stable findings than small groups such as students or app users. Differences between studies appeared to reflect mainly from methodological variation rather than true differences between population subgroups.

### 3.11. Sensitivity Analyses

**Figure 5:**
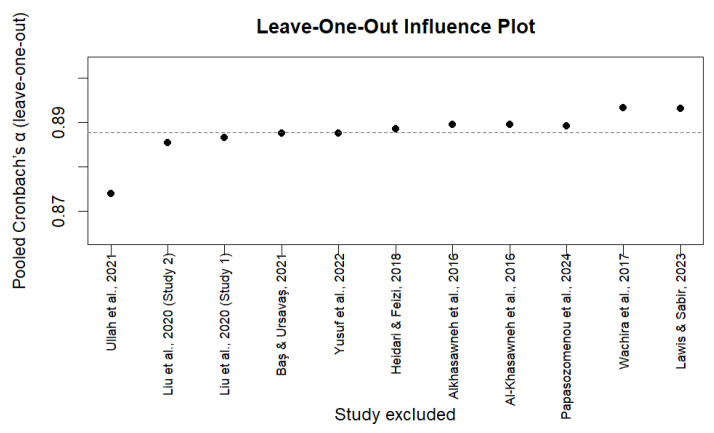
Leave-one-out influence analysis of pooled Cronbach’s α for the Breast-CAM [23,24,26,27,28,29,31,32,33,34,35].

A leave-one-out sensitivity analysis was performed to examine the robustness of the pooled internal consistency estimate [47]. Removing each study in turn led to only small changes in Cronbach’s α, which remained between 0.87 and 0.90 compared with the full-model estimate of 0.89. This indicates that no single study had a meaningful influence on the pooled result [48,49]. The substantial heterogeneity therefore likely reflects real differences between Breast-CAM versions rather than instability of the meta-analysis (Figure 5).

Each point represents the recalculated pooled reliability estimate after removal of one study from the meta-analysis. The dashed line indicates the original pooled α (0.89). Minimal variation in α across iterations (0.87–0.90) suggests that no single study unduly influenced the overall estimate, supporting the robustness of internal consistency findings.

### 3.12. Heterogeneity Exploration

High heterogeneity was observed [48] in the meta-analysis of internal consistency. Differences in item content likely contributed to this variability. Variability was observed across Breast-CAM versions in item content, scale length, and response formats. Cultural adaptation procedures also differed, and study populations and settings were heterogeneous. Because of the small number of included studies and the methodological diversity [47] of the instruments, subgroup or meta-regression analyses were not conducted. Heterogeneity was therefore examined narratively and through sensitivity analyses. These analyses showed that no individual study had a meaningful influence on the pooled estimate.

### 3.13. Certainty of Evidence

The certainty of evidence for each measurement property of the Breast-CAM was assessed using the COSMIN-modified GRADE framework. Ratings were applied at the level of the synthesized measurement property rather than individual studies. Four domains—risk of bias, inconsistency, imprecision, and indirectness—were evaluated, and downgrading occurred when any domain showed serious limitations. As recommended by COSMIN, the ceiling rule was applied, meaning the certainty rating for internal consistency could not exceed that of structural validity [10,12]. A summary of all certainty ratings is presented in Table 12.

#### 3.13.1. Internal Consistency

Reported Cronbach’s α values for the Breast-CAM were generally high [40] across culturally adapted versions. The pooled estimate was 0.89 (95% CI 0.85–0.92), exceeding commonly used thresholds. Because structural validity was insufficient or inconsistently assessed across versions, internal consistency was rated as indeterminate under COSMIN criteria. Certainty of evidence was downgraded for inconsistency—largely due to versions without structural validity assessment—and for methodological heterogeneity. In line with the COSMIN ceiling rule, the certainty of evidence for internal consistency did not exceed that for structural validity.

#### 3.13.2. Structural Validity

Evidence for structural validity was of low certainty. Only three studies used EFA and CFA to confirm factor structure [43] while most versions reported α values without testing dimensionality. Downgrades reflected risk of bias (incomplete construct testing) and inconsistency in reported models.

#### 3.13.3. Content Validity

Content validity was supported by low-certainty evidence. Twelve studies described cultural adaptation and expert review, and seven provided clear evidence of relevance and comprehensibility. Downgrades reflected indirectness (e.g., limited use of population-specific cognitive interviews) and reporting gaps, particularly the lack of I-CVI/S-CVI [14,42] indices in some studies.

#### 3.13.4. Reliability (Test–Retest)

Very low-certainty evidence suggests acceptable test–retest reliability. Only two studies reported ICC values > 0.70 [41]. Small samples, inconsistent intervals, and partial reporting led to downgrades for imprecision and inconsistency.

#### 3.13.5. Measurement Error

Measurement error evidence was of very low certainty. One study reported SEM and SDC, but without MIC values interpretability [38] was limited. Evidence was downgraded for imprecision and indirectness.

#### 3.13.6. Construct Validity/Hypothesis Testing

Low-certainty evidence supports construct validity. Four of eight studies confirmed predefined hypotheses [44] others relied on exploratory associations with demographics. Downgrades reflected inconsistency and the absence of prespecified hypotheses.

#### 3.13.7. Criterion Validity

Criterion validity evidence was rated very low. No study compared the Breast-CAM against a true gold standard, and only two studies used indirect comparators [45]. These comparators captured related aspects of awareness but did not represent an established reference standard. As a result, the evidence was downgraded for indirectness and lack of an appropriate criterion.

#### 3.13.8. Responsiveness

Low-certainty evidence suggests that the Breast-CAM is responsive to educational interventions. Three of four studies demonstrated meaningful pre–post improvements. Certainty was downgraded for indirectness, as MIC values were not established [38].

#### 3.13.9. Cross-Cultural Adaptation (Not Graded)

Cross-cultural adaptation procedures [46] were described in 14 studies. Seven followed full translation–review–pilot protocols, while others used partial or undocumented methods. Adaptation was not graded because it is a preparatory step rather than a psychometric property.

Overall, reported Cronbach’s α values for the Breast-CAM were generally high across culturally adapted versions. At the overall instrument level, internal consistency was rated as indeterminate due to insufficient or inconsistent evidence for structural validity. Evidence for structural validity, content validity, construct validity, and responsiveness was generally supported by low certainty. Evidence for reliability and measurement error was rated as very low certainty. Although cross-cultural adaptation procedures were often reported, they were not graded because they represent a preparatory step rather than a psychometric measurement property. A summary of overall ratings and certainty of evidence for each measurement property is presented in Table 13.

### 3.14. Recommendations for Use and Research

Based on the synthesized evidence, the Breast-CAM may be used selectively, depending on its intended purpose and the strength of the available measurement evidence. The Breast-CAM is most appropriate for population-level assessment, including community surveys, public health monitoring, and evaluation of awareness-raising programs. Across adaptations, internal consistency estimates were generally high [12]. However, COSMIN ratings for internal consistency were classified as indeterminate because evidence for structural validity was limited. Several studies still demonstrated responsiveness to educational interventions. These findings support its use for tracking group-level changes in awareness rather than for individual decision-making [38].

#### 3.14.1. Versions with the Strongest Empirical Support

Where rigorous measurement performance is required, priority should be given to adaptations that established structural validity and followed comprehensive cultural adaptation procedures. These include the Persian BCAM [27], Chinese BCAM [28], and Turkish BCAM [33]. These versions confirmed underlying dimensional structures (via EFA/CFA), reported strong internal consistency (α > 0.80), and demonstrated clearer methodological transparency [13,38]. They are therefore well suited for research and program evaluation contexts where robust psychometric evidence is essential.

#### 3.14.2. Versions Appropriate for Exploratory or Preliminary Use

Some adaptations—such as the Arabic [23,24,25], Swahili [26], Greek [35], and Malay intervention-based version [32]—show acceptable internal consistency and reasonable cultural adaptation [38]. However, because evidence for structural validity and reliability is limited, these versions are best used for exploratory or descriptive studies, with cautious interpretation of results [12].

#### 3.14.3. Versions Not Recommended for Individual-Level Interpretation

Adaptations with incomplete reporting or insufficient psychometric evidence—such as the Pakistani study [31], the Sudanese study [34], and the Chinese outcome-use study without psychometric re-evaluation [29]—are not recommended for applications requiring individual-level interpretation or screening decisions [12]. These versions may still be used for generating hypotheses or preliminary descriptive work.

#### 3.14.4. Limitations Applicable Across All Versions

Across the evidence base, documentation of test–retest reliability, measurement error, and interpretability thresholds was limited. No version has established criterion validity, and minimal important change values are unavailable [10]. As a result, absolute score interpretation and cross-population comparisons should be made cautiously, regardless of version.

## 4. Discussion

To our knowledge, this review is the first to systematically synthesize—and, where possible, meta-analyze—the psychometric performance of the Breast Cancer Awareness Measure (Breast-CAM) across culturally adapted versions. Previous validations have been conducted in individual settings, including Arabic [24,25], Swahili [26], Persian [27], Chinese [28,29], Turkish [33], Malay [32], Sudanese [34], and Greek contexts [35]. While these studies provide valuable insight into local adaptation, none examined whether the instrument performs consistently across populations or synthesized psychometric evidence within a unified framework. As a result, questions regarding cross-cultural comparability and overall measurement quality have remained unresolved. Patterns of breast cancer incidence and mortality vary across regions and age groups, underscoring the need for culturally sensitive awareness tools [50].

Across the included studies, internal consistency was the most frequently reported measurement property [13]. Most adaptations reported Cronbach’s α values above commonly accepted thresholds [39] and the pooled estimate reflected this pattern. These findings align with the intended conceptual structure of the Breast-CAM and suggest that reported internal consistency estimates for core awareness domains were generally high. Because structural validity was infrequently assessed, confidence in internal consistency as a measurement property cannot be clearly established under COSMIN standards [51]. Evidence for several other psychometric properties remains limited [10,51]. Test–retest reliability was rarely assessed, and measurement error and interpretability thresholds were largely unexamined. Responsiveness was evaluated in only a small number of studies [51]. Given the influence of educational exposure, cultural beliefs, and socio-behavioral factors on awareness, these gaps are understandable. They limit confidence in the instrument’s ability to perform consistently over time. The available evidence supports the use of the Breast-CAM primarily as a group-level assessment tool in community surveys and evaluations of awareness interventions [12]. At present, the evidence is insufficient to support its use in clinical decision-making, individual risk assessment, or screening.

### 4.1. Limitations of the Evidence Included in the Review

Several limitations should be considered when interpreting the findings of this review. Although extensive database and registry searches were conducted, restriction to English-language publications may have introduced language bias and resulted in the lack of locally developed or unpublished adaptations of the Breast-CAM. In addition, culturally adapted versions reported in gray literature [52], theses, or program evaluations may not have been captured, potentially under-representing adaptations from low- and middle-income settings. None of the included studies formally assessed measurement invariance [53] across language or cultural versions, limiting the ability to determine whether pooled estimates reflect equivalent constructs across populations. Pooling psychometric estimates across heterogeneous adaptations is challenging because versions differ in item content, scoring, and study design. Cronbach’s α depends on the number of items and the underlying factor structure. Because Breast-CAM adaptations varied in dimensional structure, internal consistency estimates across studies may not be directly comparable, and pooled α values should be interpreted cautiously. The body of evidence available to evaluate the Breast-CAM is also limited in scope and depth. Most validation studies relied on small, convenience samples drawn from university populations, outpatient clinics, or community education programs. This sampling approach limits generalizability, particularly to underserved or higher-risk populations in which awareness gaps may be most pronounced. Demographic characteristics such as socioeconomic status, literacy level, and access to healthcare were reported inconsistently, making it difficult to assess whether the instrument performs similarly across diverse groups.

Reporting of psychometric properties varied across the included studies. Internal consistency was almost always reported, but other properties central to COSMIN standards—such as structural validity, test–retest reliability, responsiveness, and measurement error—were often assessed inconsistently or described in limited detail [12,13]. Construct validity was frequently examined using descriptive analyses rather than predefined hypotheses or theory-driven approaches. Similar limitations have been noted in recent evaluations of outcome measurement instrument reviews, which highlight wide variation in methodological quality and recurrent shortcomings in psychometric assessment. Together, these issues reduce confidence in individual study findings and constrain the interpretation of pooled estimates, reinforcing the importance of close adherence to COSMIN guidance [51].

Adaptation procedures contributed further variability. Several studies relied on forward–back translation without incorporating cognitive interviews or direct engagement with target populations, raising concerns about conceptual equivalence across cultures. The predominance of cross-sectional designs also limits insight into temporal stability and sensitivity to change. Geographical representation was uneven, with stronger coverage in the Middle East and East Asia but limited evidence from regions with high breast cancer mortality. Collectively, these limitations constrain external validity and highlight the need for more rigorous and geographically diverse validation efforts.

### 4.2. Limitations of the Review Processes Used

Several methodological aspects of this review may influence how the findings are interpreted. Although the search strategy was comprehensive and covered multiple databases and trial registries, no psychometric evaluations beyond the peer-reviewed literature were identified. Studies that used the Breast-CAM only as an outcome measure, without assessing its measurement properties, were therefore excluded. The synthesis reflects the published psychometric evidence and may not capture unpublished or locally developed adaptations.

Limiting the review to English-language publications may have introduced language bias. Although multiple culturally adapted versions of the Breast-CAM from diverse linguistic and regional populations were included, validation studies published exclusively in local-language journals may not have been captured. This may limit the comprehensiveness of the review and should be considered when interpreting conclusions regarding global cross-cultural validity. Screening was conducted by a team of four reviewers with support from ASReview to priorities likely relevant records. While machine-learning assistance [16,54] improved efficiency, it relies on training inputs, and studies using atypical terminology may have required manual identification. Although all disagreements were resolved through consensus, the combination of automation and reviewer judgment introduces a small risk of selection bias.

The completeness of the synthesis was shaped by limitations in primary reporting. Key properties such as measurement error, temporal stability, and factorial structure were frequently missing, precluding more detailed psychometric evaluation. Planned subgroup or comparative analyses were not feasible due to insufficient or inconsistent data. These limitations do not undermine the overall conclusions but reduce the certainty with which findings can be generalized [10].

### 4.3. Implications for Practice, Policy, and Future Research

The findings of this review suggest that the Breast-CAM is well suited for population-level awareness assessment, particularly in community surveys and public health program evaluation [8]. Across multiple cultural adaptations—including Arabic [23,24], Swahili [26], Persian [27], Chinese [28], Malay [32], Turkish [33], Sudanese [34], and Greek versions [35]— reported Cronbach’s α values for symptom recognition items were generally high, [13] although evidence for structural validity was limited. The instrument can support identification of knowledge gaps, monitoring of awareness initiatives, and planning of targeted public health messaging.

Greater caution is warranted when interpreting domains related to beliefs, barriers, and help-seeking intentions. Variation in these domains likely reflects genuine contextual differences—such as stigma, cultural norms, and access to screening—rather than weaknesses in the instrument itself [55]. Policymakers should therefore avoid direct cross-country comparisons and interpret findings in relation to local sociocultural and health-system contexts.

Future research should prioritize strengthening the psychometric foundation of the Breast-CAM. Structural validity should be evaluated systematically using confirmatory factor analysis or item-response theory rather than relying on internal consistency alone. Longitudinal studies are needed to assess temporal stability and establish minimal important change values. Cultural adaptation processes should incorporate cognitive interviewing, stakeholder engagement, and multi-group invariance testing [53] to ensure conceptual equivalence beyond literal translation. Greater emphasis should be placed on under-represented populations—including older adults, low-literacy groups, rural communities, and women facing structural barriers to screening—to ensure the instrument remains relevant and equitable across diverse settings. Table 14 summarizes the key priorities for strengthening the psychometric evaluation and future application of the Breast-CAM identified in this review.

## 5. Conclusions

This systematic review and meta-analysis is the first comprehensive synthesis of psychometric evidence on the Breast Cancer Awareness Measure across culturally adapted versions. The findings suggest that reported internal consistency estimates for the Breast-CAM are generally high across different settings, supporting its use in population-level assessments and public health program evaluation. Evidence for other measurement properties remains limited and inconsistently reported. Structural validity, test–retest reliability, responsiveness, and measurement invariance have not been systematically examined. Until these gaps are addressed, the Breast-CAM should be used with caution for purposes beyond group-level assessment. Further psychometric research using rigorous and longitudinal designs is needed to support broader application of the instrument.

## Figures and Tables

**Table 1:** Search sources, coverage, search dates, and records retrieved.

Host/Platform	Database/Register	Coverage Period	Date Searched	Records Retrieved
Wiley	Cochrane Library	January 2010–15 October 2025	16 September 2025	37 (6 reviews, 1 protocol, 30 trials)
EBSCOhost	CINAHL/EBSCO	January 2010–15 October 2025	16 September 2025	144
Elsevier	Embase	January 2010–15 October 2025	15 September 2025	377
Google	Google Scholar + University Repositories	January 2010–15 October 2025	22 September 2025	101 + 1
NLM	PubMed	January 2010–15 October 2025	15 September 2025	204 (filtered: free full text)
Elsevier	Scopus	January 2010–15 October 2025	16 September 2025	32
Clarivate	Web of Science	January 2010–15 October 2025	15 September 2025	505
ClinicalTrials.gov	ClinicalTrials.gov	January 2010–15 October 2025	17 September 2025	46
WHO ICTRP	WHO ICTRP	January 2010–15 October 2025	13 October 2025	0
ISRCTN Registry	ISRCTN	January 2010–15 October 2025	14 October 2025	0
EU-CTR	EU Clinical Trials Register	January 2010–15 October 2025	15 October 2025	0

Note: Total database records = 1446; total registry records = 46. Searches were conducted between 15 September and 15 October 2025. The search time frame was January 2010 to 15 October 2025. The search strategy was reported according to PRISMA-S [7].

**Table 2:** Summary of Measurement Properties (SOMP) and COSMIN Rating Criteria.

Measurement Property	Rating	COSMIN Criteria Used to Assign Rating
Content validity	Sufficient (+)	Relevant, comprehensive, and understandable items (expert review or user testing).
	Insufficient (−)	Inadequate relevance, comprehensiveness, or comprehensibility.
	Indeterminate (?)	Unclear, incomplete, or not reported procedures.
Structural validity	Sufficient (+)	CFA or IRT/Rasch models with acceptable fit (e.g., CFI/TLI ≥ 0.95; RMSEA ≤ 0.06; SRMR ≤ 0.08).
	Insufficient (−)	EFA without CFA, inadequate model fit, or no structural evaluation.
	Indeterminate (?)	Structural testing reported but inadequately described.
Internal consistency	Sufficient (+)	Cronbach’s α 0.70–0.95 with confirmed structural validity.
	Insufficient (−)	α < 0.70 or > 0.95, or no evidence of dimensionality.
	Indeterminate (?)	α reported without confirmed structural validity.
Reliability (test–retest/ICC/κ)	Sufficient (+)	ICC or weighted κ ≥ 0.70 with appropriate retest interval.
	Insufficient (−)	ICC/κ < 0.70 or inappropriate methodology.
	Indeterminate (?)	Reliability assessed but insufficient reporting.
Measurement error	Sufficient (+)	SDC or LoA < Minimal Important Change (MIC).
	Insufficient (−)	SDC/LoA ≥ MIC or clear imprecision.
	Indeterminate (?)	SEM/SDC reported but MIC not defined.
Construct validity (hypothesis testing)	Sufficient (+)	≥75% of predefined hypotheses confirmed.
	Insufficient (−)	Fewer than 75% of hypotheses confirmed, or contradictory findings
	Indeterminate (?)	No predefined hypotheses.
Criterion validity	Sufficient (+)	Correlation with gold standard ≥ 0.70, or AUC ≥ 0.70.
	Insufficient (−)	Inadequate comparator or correlation/AUC < 0.70.
	Indeterminate (?)	Comparator unclear or insufficiently reported.
Responsiveness	Sufficient (+)	≥75% change hypotheses confirmed, or AUC ≥ 0.70.
	Insufficient (−)	No expected change or poor design.
	Indeterminate (?)	Incomplete change data, or no MIC.
Cross-cultural adaptation	Sufficient (+)	Forward–backward translation, expert review, and pilot/cognitive testing performed.
	Insufficient (−)	Key adaptation steps missing).
	Indeterminate (?)	Adaptation procedures missing or incomplete.

Notes: Internal consistency ratings were considered valid only when structural validity was established. Pooled Cronbach’s α values are presented descriptively and were not interpreted as evidence of sufficient internal consistency without confirmed structural validity. Responsiveness was judged relative to predefined change hypotheses or AUC thresholds. Cross-cultural adaptation reflects linguistic and contextual adequacy and is evaluated separately from psychometric properties. + = sufficient; − = insufficient; ? = indeterminate. Ratings were assigned according to COSMIN criteria for good measurement properties and reflect whether studies met predefined psychometric thresholds. Abbreviations: CFI—Comparative Fit Index; TLI—Tucker–Lewis Index; RMSEA—Root Mean Square Error of Approximation; SRMR—Standardized Root Mean Square Residual; ICC—Intraclass Correlation Coefficient; κ—Cohen’s kappa; SEM—Standard Error of Measurement; SDC—Smallest Detectable Change; LoA—Limits of Agreement; MIC—Minimal Important Change; AUC—Area Under the Receiver Operating Characteristic Curve.

**Table 3:** Decision rules for applying the COSMIN-modified GRADE approach.

GRADE Factor	Downgrading Level	Decision Rule
Risk of bias	0/−1/−2	Downgraded based on COSMIN Risk of Bias assessments. One level was downgraded when most evidence came from studies rated as doubtful. Two levels were downgraded when serious methodological limitations were present across studies.
Imprecision	0/−1/−2	Downgraded based on total sample size contributing to the evidence: ≥100 = no downgrade; 50–99 = −1; <50 = −2.
Inconsistency	0/−1	Downgraded when substantial unexplained heterogeneity was present or when results varied widely across studies.
Indirectness	0/−1	Downgraded when study populations, cultural contexts, or Breast-CAM versions differed from the intended context of use.
Ceiling rule	—	In accordance with COSMIN guidance, internal consistency was not rated higher than structural validity when evidence for dimensionality was insufficient.

**Table 4:** Recommended use of the Breast-CAM based on certainty of evidence.

Certainty of Evidence (COSMIN–GRADE)	Interpretation	Appropriate Use of Breast-CAM	Not Recommended Use
High/Moderate	Measurement properties adequately supported	Population awareness surveys; public health monitoring; evaluation of awareness interventions	—
Low	Some supporting evidence but important limitations	Exploratory research and group-level comparisons (interpret with caution)	Individual clinical decisions
Very low	Insufficient psychometric support	Descriptive research only	Clinical decision-making; individual risk assessment; monitoring change in individual patients

**Table 5:** Characteristics of Breast-CAM versions and psychometric evaluations.

Instrument Version	Type of Measure	Study Participants (Original Adaptation)	Setting	Subscales/Items	Response Format	Translation/Adaptation Method	Psychometric Properties Assessed	Reference
BCAM v1 (Original UK)	Self-administered awareness questionnaire	Women attending NHS Breast Screening Programme	Community screening clinics	Symptoms (8); Risk factors (11); Screening; Barriers	Yes/No/Don’t know; Likert (barriers)	Developed in English	κ, content validity, test–retest, responsiveness	Linsell et al. (2010) [8]
BCAM v1 (Intervention format)	Same as BCAM v1	Older women (67–70 yrs)	Community, PEP trial	Same as original BCAM	Same as original	No translation (English)	Responsiveness, content validity, hypothesis testing	Forbes et al. (2011) [21]
Swahili BCAM (Kenya)	BCAM-based knowledge questionnaire	Kenyan women attending education interventions	Community screening & education events	BCAM-derived symptoms and risk-factor items	Categorical (True/False/Don’t know)	BCAM-informed questionnaire; translation and cultural adaptation procedures not reported	Responsiveness only (pre–post change)	Kisuya et al. (2015) [22]
Arabic BCAM (Oman)	Culturally adapted questionnaire	Arab women ≥20 yrs	Health centers & community	Symptoms; Risk factors; Barriers	Yes/No/Don’t know; Likert	WHO translation steps + pilot	α, inter-rater reliability, CVI, HT, floor/ceiling, CCA	Alkhasawneh et al. (2016) [23]; Al-Khasawneh et al. (2016) [24]
Modified Arabic “Breast Module”	Module of BCAM adapted culturally	Arab women	Community health settings	Modified BCAM symptoms and risk modules	Yes/No/Don’t know	Translation + tailoring	α, split-half, test–retest, responsiveness	Al Khasawneh et al. (2017) [25]
Swahili BCAM (Kenya)	Culturally adapted questionnaire	Kenyan women (young adults and general population)	Community, research field sites	Symptoms: Risk factors (modified); Screening; Barriers	Yes/No/Don’t know	Forward–backward, expert review, FGDs	α, EFA, CVI, HT, floor/ceiling, cross-cultural adaptation	Wachira et al. (2017) [26]
Persian BCAM	Translated & adapted BCAM	Persian-speaking women	Primary healthcare settings	Symptoms; Risk factors; Screening; Barriers	Yes/No/Don’t know	Forward–backward, panel review, pilot test	α, ICC, κ, EFA, CFA, ROC/AUC, SEM, SDC	Heidari & Feizi (2018) [27]
Chinese BCAM (C-BCAM)	Translated & adapted BCAM	Chinese women (19–67 yrs)	Community & outpatient clinics	Symptoms; Risk factors; Screening; Barriers	Yes/No/Don’t know	Brislin forward–backward; cognitive interviews	α, ICC, EFA, CFA, CVI, ROC/AUC	Liu et al. (2020) [28]; Liu et al. (2020) [29]
Malay BCAM (B-CAM-M)	Translated BCAM	Malay-speaking women ≥18 yrs	Community and health centers	Symptoms; Risk factors; Screening; Barriers	Yes/No/Don’t know	WHO forward–backward	α, ICC, CVI, linguistic validity	Htay et al. (2020) [30]
BCAM (CR-UK version, Pakistan)	BCAM used as awareness outcome questionnaire	Adult female patients attending outpatient departments	Hospital outpatient clinics	Symptoms; Risk factors; Screening; Barriers	Yes/No/Don’t know	Direct use of BCAM with Urdu translation; adaptation procedures not fully described	α, content validity, hypothesis testing (descriptive associations)	Ullah et al. (2021) [31]
Malay BCAM (App-adapted)	BCAM adapted for mobile app	Malay women in app-based learning	Community/mobile health	Symptoms; Screening; Barriers	Yes/No/Don’t know	Cultural tailoring for digital content	α, responsiveness, content adaptation	Yusuf et al. (2022) [32]
Turkish BCAM	Translated & culturally adapted BCAM	Turkish women	Community women’s health settings	Symptoms; Risk factors; Screening; Barriers	Yes/No/Don’t know	WHO translation method + pilot	α, ICC, κ, CFA, CVI, HT	Baş & Ursavaş (2023) [33]
Sudanese BCAM	Culturally adapted BCAM	Sudanese females in Omdurman	Community health outreach	Symptoms; Risk factors	Yes/No/Don’t know	Cultural modification & expert review	α, content validity, hypothesis testing, CCA	Lawis & Sabir (2023) [34]
Greek BCAM	Transcultural adaptation	Greek women in rural border area	Community clinics	Symptoms; Risk factors; Screening	Yes/No/Don’t know	Forward backward + expert panel + pilot	α, exploratory construct validity, content validity	Papasozomenou et al. (2024) [35]
Palestinian BCAM	Arabic-translated modified BCAM	Palestinian women (18–74 yrs)	Hospitals, PHCs, public venues	Symptoms (13 items); Barriers	Yes/No/Don’t know; Likert	Translation + cultural adaptation	α, CVI, content adaptation	Elshami et al. (2025) [36]

Footnotes: α: Cronbach’s alpha; ICC: Intraclass correlation coefficient; κ: Cohen’s kappa; CVI: Content validity index; HT: Hypothesis testing; EFA: Exploratory factor analysis; CFA: Confirmatory factor analysis; ROC: Receiver operating characteristic; AUC: Area under the curve; SEM: Standard error of measurement; SDC: Smallest detectable change; CCA: Cross-cultural adaptation; FGDs: Focus group discussions; PHC: Primary healthcare center.

**Table 6:** Interpretability and distributional characteristics of Breast-CAM versions.

OMI (Version)	Study	Distribution of Scores	Missing Data (%)	Floor/Ceiling Effects	Scores/Change Scores Available	MIC/MID Reported
BCAM v1 (original English)	Linsell et al., 2010 (UK) [8]	Awareness score 0–3 based on 3 components. At baseline only 2% were fully aware (score 3/3); 27% scored 2, 44% scored 1 and 27% scored 0. Item-level recognition ranged from very high for breast lumps to very low for non-lump signs and age-related risk.	For the three core items, non-response around 1–6%; awareness scores calculable for 95% of women.	Marked ceiling for lump symptoms and floor for age-related risk. No formal % floor/ceiling reported.	Cross-sectional comparisons of total awareness score across subgroups (age, ethnicity, deprivation) and between cancer experts vs. general women; BCAM able to discriminate groups with different expected awareness levels.	Not reported.
BCAM v1 (original English)	Forbes et al., 2011 (UK) [21]	Baseline overall awareness very low (≈1.8–3.4% fully aware). At 2 years: 6% fully aware in usual care vs. 21% in PEP group. Component distributions shown by time-point.	Exact % not given; follow-up response ≈ 78–80% at 2 years	No formal floor/ceiling analysis; lump awareness was near ceiling, while age-related risk remained near floor (~10% correct)	Repeated measures at baseline, 1 month, 6 months, 1 year and 2 years provide a clear change-score trajectory.	Not reported.
Kenyan adapted BCAM	Kisuya et al., 2015 (Kenya) [22]	Knowledge score (0–16) created from BCAM-based items. Mean total score increased from roughly low–moderate at baseline to substantially higher post-intervention (mean change ≈ +2.8 points on 0–16 scale, *p* < 0.001). Item-level proportions of correct answers also increased.	Sample of *n* = 532 analyzed; item-level missing data not detailed, suggesting minimal missingness.	No explicit total-score floor/ceiling analysis. Most items showed mid-range baseline knowledge with clear post-intervention improvement; some cure-related items approached higher scores post-education, but not extreme ceiling.	Pre/post scores: mean change +2.80 (95% CI 2.38–3.22), *p* < 0.001, demonstrating clear responsiveness to education.	Not reported.
Arabic BCAM (Oman, mixed methods)	Alkhasawneh et al., 2016—“I do not even say ‘it’” (Oman) [23]	Descriptive item-level distributions of awareness: many women correctly recognized breast lumps, but awareness of several non-lump signs, risk factors and screening recommendations was low. No summed BCAM score was reported.	Large quantitative sample (*n* ≈ 1370); completion was high, but exact % missing per item not provided.	Floor/ceiling not analyzed formally. Pattern suggests low scores (floor-like) for several warning signs and risk-factor items, with no evidence of overall ceiling.	Only cross-sectional descriptive percentages and regression analyses (associations with demographics and beliefs); no total or subscale scores and no change scores.	Not reported.
Arabic-BCAM-A (transcultural adaptation)	Al-Khasawneh et al., 2016—Int Nurs Rev (Oman) [24]	For warning-sign and risk-factor subscales, item-level correct-answer percentages presented. Total subscale scores used in internal-consistency analyses, but no overall mean/median totals are displayed for the sample.	Very high acceptability: 98.7% completed the questionnaire; item-level missing data negligible but not tabulated.	Authors explicitly report that all floor and ceiling effects for subscales were <15%, indicating absence of serious floor/ceiling problems.	Cross-sectional scores for risk-factor and warning-sign subscales used for internal-consistency and criterion-validity analyses (correlation with an external awareness indicator), but no pre/post or longitudinal change scores.	Not reported.
Modified Arabic BCAM “breast module”	Alkhasawneh et al., 2017 (Oman) [25]	Small interventional sample (*n* = 53). The BCAM-derived “breast module” was scored and used to evaluate a tailored education program. Scores increased meaningfully from pre- to post-education (statistically significant improvement), but detailed means/SDs for the total score are not fully reported.	No formal description of missing data; given small *n* and direct administration, missingness appears minimal.	Floor/ceiling not assessed. Baseline scores suggest low–moderate awareness with scope for improvement; post-program scores do not appear to reach ceiling.	Pre–post comparisons for the BCAM-based module (including split-half and test–retest reliability) demonstrate responsiveness to the culturally tailored program.	Not reported.
Swahili BCAM (Kenya)	Wachira et al., 2017 (Kenya) [26]	Distributions presented mainly as percentages of women correctly identifying symptoms and risk factors and as distributions of Likert-type responses for barriers. Total or subscale BCAM scores are not the focus; instead, factor-based scales are examined psychometrically.	In the survey sample (*n* = 1061), completion was very high; the paper does not present explicit item-level missing-data percentages.	No formal floor/ceiling analyses. Many symptom-awareness items show moderate recognition; several barrier items cluster at “agree”, but without numerical floor/ceiling summaries.	Factor-scale scores (derived from EFA) are used for construct-validity tests and group comparisons, but change scores are not reported.	Not reported.
Persian BCAM	Heidari & Feizi, 2018 (Iran) [27]	For warning-signs subscale (11 items), mean (SD) total score 3.85 (3.02) in general women vs. 7.96 (3.00) in medical/clinical experts (0–11 scale). Latent-class analysis identified three awareness classes: high (≈13%), moderate (≈61%) and low (≈26%).	Overall *n* = 1078; respondents with missing core BCAM items were excluded. Exact percentage of missing BCAM items is not detailed but appears low.	Explicitly evaluated: 10% of women scored the minimum of 0 and 14% scored the maximum of 11. Both below the 15% COSMIN threshold → no important floor/ceiling effect.	Cross-sectional comparisons of total scores across expert/general-population groups and across covariates; ROC curves used to show discriminant ability. SEM and SDC reported, supporting interpretability of change in future interventional work.	SEM = 3.96; SDC = 10.98; MIC/MID not estimated
Chinese BCAM—psychometric version (C-BCAM)	Liu et al., 2020 (BMJ Open, China) [28]	Overall breast-cancer awareness showed a median total C-BCAM score around the mid-70 s on a 0–100 transformed scale (e.g., median ≈ 76.5, IQR roughly ~69–84). Domain medians were in the moderate range for symptoms, barriers and risk-factors subscales.	Data judged skewed; analyses based on medians and IQRs. Item non-response was very low; exact percentages not provided.	The study does not report formal floor/ceiling percentages. From the reported medians and IQRs, scores cluster away from minimum and maximum, suggesting no major floor/ceiling problem.	Cross-sectional total and domain scores compared across socio-demographic groups and used in EFA/CFA. No longitudinal change scores were collected.	Flesch reading-ease score = 87.9 (indicates very easy comprehension) 91% of participants found the scale easy to read 96% reported the items were not upsetting
Chinese BCAM—awareness/factors study (C-BCAM)	Liu et al., 2020 (China) [29]	C-BCAM used as outcome; continuous total score analyzed with median and IQR. Overall awareness was in the mid-range (total median around mid-70 s/100), with higher scores in screening-eligible and previously screened women. Domain scores for symptoms, barriers and risk factors showed similar mid-range distributions.	Missing data not described in detail; analyses suggest near-complete data for included participants.	No explicit floor/ceiling analyses. Score distributions (medians well above 0 and below maximum) indicate no extreme floor/ceiling.	Cross-sectional: total C-BCAM score compared across socio-demographic, screening-behavior and attitudinal groups (e.g., different screening preferences). No change scores.	Not reported.
Malay BCAM-M (translation/validation)	Htay et al., 2020 (Malaysia) [30]	No overall BCAM-M total or subscale score statistics (mean/median) reported. Results are presented mainly as percentages of correct responses for symptoms, risk-factor and screening items.	Sample *n* = 251; completion high. Item-level missing data not quantified.	Floor/ceiling not analyzed; some items show high correct-answer rates (near-ceiling behavior), others very low, but without formal reporting.	Cross-sectional design: BCAM-M scores used for reliability and validity analyses only (α, ICC, CVI). No change scores.	Not reported.
BCAM	Ullah et al., 2021 (Pakistan) [31]	Awareness described only as percentages of women answering individual items correctly (e.g., proportion recognizing each symptom or risk factor). No summed BCAM score or distribution of total scores is presented.	*n* = 430; paper does not report missing data percentages; analyses imply near-complete responses.	Floor/ceiling not evaluated. Several core-symptom items have high recognition, while some risk-factor items have very low recognition.	BCAM is used purely as a descriptive outcome and for hypothesis testing with socio-demographic variables; no composite scores or change scores.	Not reported.
Malay BCAM-M used in mobile-app intervention	Yusuf et al., 2022 (Malaysia) [32]	BCAM-M-based knowledge scores compared pre- and post-intervention in the app group. The paper reports a statistically significant improvement in awareness after using the BrAware app but does not provide detailed means/medians for a global score in a way that can be extracted for interpretability.	Small sample (*n* = 41). Missing data is not reported; analyses imply full paired data for those retained.	Floor/ceiling not examined. Baseline knowledge appears modest, with notable improvement post-intervention; scores do not appear to reach ceiling.	Pre–post comparisons of BCAM-M items and composite awareness indices show significant gains in awareness, demonstrating responsiveness of the tool to a mobile-app intervention.	Not reported.
Turkish BCAM-Tr	Baş et al., 2023 (Turkey) [33]	Respondents receive 0–3 total score. The paper reports item-level correct-answer percentages and uses the 0–3 total score for reliability and construct-validity analyses, but detailed descriptive statistics (means/medians) of the total score are not given.	*n* = 552; acceptability good. Explicit missing data percentages are not supplied.	Floor/ceiling for the total 0–3 score is not reported. Item-level data indicate especially low awareness for age-related risk, with higher awareness for lump symptoms.	Cross-sectional. Total BCAM-Tr score used in test–retest and known-groups comparisons; no longitudinal change or responsiveness data.	Not reported.
Sudanese modified BCAM	Lawis & Sabir, 2023 (Sudan) [34]	Awareness reported as percentages of correct answers to BCAM-based items (symptoms, risk factors, screening behaviors). No total BCAM-derived score or summary distribution provided.	*n* = 385, community sample; the article does not specify missing data percentages.	Floor/ceiling not assessed. Many non-lump symptoms and several risk-factor items show low recognition, suggesting a tendency towards lower scores without true floor effects being quantified.	Cross-sectional only; used for describing awareness levels and associations with socio-demographic variables. No change scores.	Not reported.
Greek modified BCAM	Papasozomenou et al., 2024 (Greece) [35]	Results are presented as category distributions for each closed-ended item (e.g., “strongly disagree” to “strongly agree” for risk-factor items; yes/no/don’t-know for symptoms). No global BCAM score or subscale totals are calculated.	*n* = 110; all questionnaires returned. Item-level missing data not described but implied to be minimal.	No formal floor/ceiling analysis. Several less common warning signs and risk-factors show high “don’t know” or incorrect responses (low awareness), while classic signs (lump) are closer to ceiling.	BCAM-based items used descriptively to profile awareness and as the basis for a reliability estimate (Cronbach’s α for the risk-factor block). No pre/post or longitudinal change scores.	Not reported.
Modified Arabic BCAM (Palestinian)	Elshami et al., 2025 (Palestine) [36]	Uses a modified, Arabic-translated BCAM with 13 symptom items plus barrier questions. Awareness presented mainly as proportions of women correctly identifying individual symptoms and barriers. No total BCAM score distribution is reported for either screening-eligible or ineligible groups.	Large nationwide sample (*n* = 2796). The paper does not detail item-level missing data percentages; questionnaire completion appears very high.	Floor/ceiling effects for the symptom scale are not calculated; some classic symptoms approach high recognition, while several others remain low, indicating a mixed pattern without formal thresholds.	BCAM-based items form part of a broader survey comparing screening-eligible vs. -ineligible women. Analyses focus on group differences in awareness and care-seeking, not on BCAM change scores or longitudinal follow-up.	Not reported.

**Table 7:** Feasibility Characteristics of Breast-CAM Versions.

OMI (Version)	Type & Ease of Administration	Length of Instrument	Response Options	Completion Time	Required Mental/Physical Ability	Ease of Score Calculation	Copyright
BCAM v1 (original English)—Linsell 2010 [8]	Self-administered paper questionnaire reported as easy to complete in screening settings.	Full BCAM: symptoms, age-risk, help-seeking, risk factors + 3-item awareness score.	Yes/No/Don’t know; single-answer items.	Not reported.	Basic reading; no physical demands.	3-item awareness score summed (0–3); other outcomes are % correct.	Cancer Research UK (CRUK).
BCAM v1 (outcome measure)—Forbes 2011 [21]	Mailed/clinic self-completion; good feasibility across follow-ups.	Same as [8] Linsell 2010.	Yes/No/Don’t know.	Not reported.	Older adults completed independently.	Full-awareness score 0–3; easily reproduced across time points.	CRUK.
Adapted BCAM (Kenya)—Kisuya 2015 [22]	Interviewer-administered; suitable for low-literacy settings.	Adapted BCAM knowledge block; 0–16 summary.	True/False/Don’t know.	Not reported.	Responds verbally to simple questions.	Simple summation (0–16); pre/post change easy to compute.	BCAM credited to CRUK.
Arabic BCAM (Oman, mixed-methods)—Alkhasawneh 2016 [23]	Structured questionnaire; mode mixed (self/interviewer).	BCAM-based symptoms, risk-factor, screening sections.	Yes/No/Don’t know.	Not reported.	Basic literacy or oral responding.	No total score used; item-level % calculations only.	BCAM credited to CRUK.
Arabic-BCAM-A (transcultural)—Al-Khasawneh 2016 [24]	Paper, mostly self-completed; very high completion.	Full Arabic adaptation.	Yes/No/Don’t know; some Likert.	Not reported.	Requires basic Arabic reading.	Subscales summed; no complex steps.	Permission noted; CRUK.
Modified Arabic BCAM “Breast Module”—Alkhasawneh 2017 [25]	Administered around an education program; high acceptability.	Shortened BCAM module.	Yes/No/Don’t know.	Not reported.	Basic literacy; support available.	Simple summed scores for pre-post change.	BCAM acknowledged.
Swahili BCAM—Wachira 2017 [26]	Interviewer-administered (household & clinic); clear comprehension.	BCAM-based, 7 domains.	Yes/No/Don’t know; Likert.	Not reported.	Interview format reduces literacy demands.	Domain scores and factor scores; straightforward.	Based on BCAM, CRUK cited.
Persian BCAM—Heidari & Feizi 2018 [27]	Self-administered in community/clinical settings.	Warning-signs 11-item scale + risk-factors/barriers.	Yes/No/Don’t know; Likert.	Not reported.	Basic reading in Persian.	Summed subscales (0–11, etc.); easy for routine use.	CRUK acknowledged.
Chinese BCAM (psychometric C-BCAM)—Liu 2020 [28]	Self-administered; high acceptability, good readability.	C-BCAM full domains; 0–100 scaled totals.	Yes/No/Don’t know; Likert.	Not reported.	Basic literacy; no special demands.	Raw sums transformed to 0–100; transparent procedure.	BCAM used with permission.
Chinese C-BCAM (outcome study)—Liu 2020 [29]	Self-administered paper survey; described as easy to understand.	Full C-BCAM.	Yes/No/Don’t know; Likert.	Not reported.	Adults with basic literacy.	Uses defined total/domain scores; simple summation & scaling.	CRUK and Chinese validation cited.
Malay BCAM-M (validation)—Htay 2020 [30]	Interviewer or self-completion; understandable across ethnic groups.	Malay BCAM-M (symptoms, risk, screening).	Yes/No/Don’t know; categorical.	Not reported.	Literacy needs reduced with interviewer mode.	Item-level % or simple sum; no complex scoring.	BCAM used with permission.
BCAM (CRUK version) as outcome—Ullah 2021 [31]	Structured questionnaire, likely interviewer-administered in clinics.	Standard BCAM blocks.	Yes/No/Don’t know.	Not reported.	Verbal answering sufficient.	Only item-level % used; minimal scoring.	BCAM credited to CRUK.
Malay BCAM-M (BrAware app)—Yusuf 2022 [32]	Self-administered (paper/electronic) in app-based study.	BCAM-M awareness items pre/post.	Yes/No/Don’t know.	Not reported.	Needs basic literacy & smartphone familiarity.	Summary of correct items; easy pre-post change.	Derived from BCAM; CRUK cited.
Turkish BCAM-Tr—Baş 2023 [33]	Self-administered paper questionnaire; good feasibility.	Full BCAM adaptation.	Yes/No/Don’t know; MCQ.	Not reported.	Basic Turkish literacy.	Total score 0–3 + subscales; simple sums.	CRUK acknowledged.
Sudanese modified BCAM—Lawis & Sabir 2023 [34]	Interviewer-administered; suitable for low-literacy communities.	Modified BCAM items + screening block.	Yes/No/Don’t know; frequency.	Not reported.	Oral responding: minimal literacy required.	Item-level %; no total score.	BCAM cited; adapted non-commercially.
Greek modified BCAM—Papasozomenou 2024 [35]	Paper questionnaire at public events; good completion.	Eight blocks (symptoms, confidence, help-seeking, age, screening, risk).	Open-ended + yes/no/don’t know + Likert.	Not reported.	Reading or oral response; accessible to general adults.	Frequencies reported; no unified score.	BCAM credited to CRUK.
Modified Arabic BCAM (nationwide)—Elshami 2025 [36]	Interviewer-administered via tablets (Kobo Toolbox); scalable across regions.	13 symptom items + barriers + socio-demographics.	Time-to-seek-help categories; Yes/No barriers.	Not reported.	Oral responding; broad suitability.	Proportions per response category; no total score.	BCAM origin acknowledged; academic use.

**Table 8:** Characteristics of included studies evaluating Breast-CAM measurement properties.

Measure/Instrument Version	Author; Year	Country	Sample Description	Level of Adaptation	N	Study Design	Rater	Psychometric Properties Reported
BCAM v1 (original)	Linsell et al., 2010 [8]	UK	Adult women, community/breast screening	Original development	292 (acceptability), 576 (sensitivity), 167 (test–retest)	Cross-sectional	Self-report	Scale development, test–retest, construct validity
BCAM v1	Forbes et al., 2011 [21]	UK	Older women (≥67), PEP RCT	Direct use	867, 202 (after PEP)	RCT	Self-report	Responsiveness to intervention
Adapted BCAM	Kisuya et al., 2015 [22]	Kenya	Women in community screening/education	Contextual adaptation	532	Intervention	Interview-administered	Responsiveness only (pre–post change); no psychometric validation
Arabic BCAM	Alkhasawneh et al., 2016 [23]	Oman	Omani community women	Direct use of Arabic-BCAM-A	1372 (quantitative), 19 (qualitative)	Cross-sectional	Self-report	Awareness outcomes; no validation
Arabic-BCAM-A	Al-Khasawneh et al., 2016 [24]	Oman	Omani community sample	Full transcultural adaptation	972	Cross-sectional	Self-report	CVI, internal consistency, test–retest, construct validity
Arabic BCAM “breast module”	Alkhasawneh et al., 2017 [25]	Oman	Women in tailored education	Modified instrument	53	Intervention	Self-report	Responsiveness only
Swahili BCAM	Wachira et al., 2017 [26]	Kenya	Women in screening/clinic	Transcultural adaptation + validation	1061	Cross-sectional	Self-report	Internal consistency, factor structure
Persian BCAM	Heidari & Feizi, 2018 [27]	Iran	Iranian community women	Transcultural adaptation + validation	1078	Cross-sectional	Self-report	CFA/EFA, Cronbach α, test–retest
C-BCAM	Liu et al., 2020 [28]	China	Community + institutional samples	Adaptation + comprehensive validation	328	Cross-sectional	Self-report	CFA, internal consistency, test–retest
C-BCAM	Liu et al., 2020 [29]	China	Female students	Direct use of validated C-BCAM	274	Cross-sectional	Self-report	No new psychometrics
BCAM-M	Htay et al., 2020 [30]	Malaysia	Malaysian community sample	Transcultural adaptation + validation	251	Cross-sectional	Self-report	Internal consistency, factor structure
BCAM (CR-UK)	Ullah et al., 2021 [31]	Pakistan	OPD female patients	Direct use	430	Cross-sectional	Interview-administered	Descriptive awareness outcomes; hypothesis testing only (no psychometric validation)
BCAM-M (BrAware)	Yusuf et al., 2022 [32]	Malaysia	App users ≥18	Direct use of validated BCAM-M	41	Pre–post intervention	Self-report (digital)	Responsiveness; no validation
BCAM-Tr	Baş et al., 2023 [33]	Turkey	Turkish women	Full transcultural adaptation + validation	552	Cross-sectional	Self-report	Cronbach α, test–retest, factor structure
Modified BCAM	Lawis & Sabir, 2023 [34]	Sudan	Community women	Contextual adaptation	385	Cross-sectional	Interview-administered	Internal consistency (Cronbach’s α only); no structural validation
Modified Greek BCAM	Papasozomenou et al., 2024 [35]	Greece	Women in rural/border region	Translation + modification	110	Cross-sectional	Self-report	Initial psychometric results (limited)
Modified Arabic BCAM	Elshami et al., 2025 [36]	Palestine	National adult female sample	Modified instrument	2796 (628 eligible group; 2168 ineligible group)	Cross-sectional	Self-report	No psychometric validation reported

**Table 9:** COSMIN ratings for measurement properties of Breast-CAM versions across included studies.

Study/PROM Version	Structural Validity	Internal Consistency	Cross-Cultural Adaptation	Reliability	Measurement Error	Criterion Validity	Hypothesis Testing	Responsiveness
Al-Khasawneh et al., 2016 (Arabic-BCAM-A, INR) [24]	Inadequate (0)	Doubtful (1+)	Very Good (3+)	Inadequate (0)	Inadequate (0)	Adequate (1+)	Adequate (1+)	Inadequate (0)
Alkhasawneh et al., 2016 (APJCP Arabic-BCAM) [23]	Inadequate (0)	Doubtful (1+)	Adequate (1+)	Inadequate (0)	Inadequate (0)	Adequate (1+)	Doubtful (1+/1−)	Inadequate (0)
Wachira et al., 2017 (Swahili-BCAM) [26]	Adequate–Very Good (2+)	Adequate (2+/1−)	Very Good (3+)	Inadequate (0)	Inadequate (0)	Inadequate (0)	Adequate (2+)	Inadequate (0)
Heidari & Feizi, 2018 (Persian-BCAM) [27]	Very Good (3+)	Very Good (3+)	Very Good (3+)	Very Good (2+)	Adequate (1+)	Doubtful (0)	Very Good (3+)	Adequate (1+)
Liu et al., 2020 (C-BCAM) [28]	Very Good (2+)	Very Good (3+)	Very Good (3+)	Adequate (1+)	Inadequate (0)	Inadequate (0)	Adequate (2+)	Inadequate (0)
Liu et al., 2020 (C-BCAM outcome study) [29]	Doubtful (0)	Doubtful (1+)	Adequate (1+)	Inadequate (0)	Inadequate (0)	Inadequate (0)	Adequate (1+)	Inadequate (0)
Ullah et al., 2021 (BCAM/CR-UK) [31]	Inadequate (0)	Doubtful (1+)	Adequate (1+)	Inadequate (0)	Inadequate (0)	Inadequate (0)	Doubtful (?)	Inadequate (0)
Yusuf et al., 2022 (BCAM-M) [32]	Inadequate (0)	Doubtful (1+)	Adequate (1+)	Inadequate (0)	Inadequate (0)	Inadequate (0)	Adequate (1+)	Adequate (1+)
Baş & Ursavaş, 2023 (BCAM-Tr) [33]	Very Good (1+)	Very Good (3+)	Very Good (3+)	Very Good (2+)	Inadequate (0)	Inadequate (0)	Very Good (3+)	Inadequate (0)
Lawis & Sabir, 2023 (Modified Arabic-BCAM) [34]	Inadequate (0)	Doubtful (1+)	Adequate (1+)	Inadequate (0)	Inadequate (0)	Inadequate (0)	Doubtful (?)	Inadequate (0)
Papasozomenou et al., 2024 (Greek BCAM) [35]	Inadequate (0)	Doubtful (1+)	Adequate (1+)	Inadequate (0)	Inadequate (0)	Inadequate (0)	Doubtful (?)	Inadequate (0)

Note: COSMIN methodological qualityratings: + = sufficient; - = insufficient; ? = indeterminate.

**Table 10:** Reported measurement properties of Breast-CAM versions.

Instrument Version	Citation	Structural Validity	Internal Consistency	Reliability	Measurement Error	Construct Validity (Hypothesis testing)	Criterion Validity	Responsiveness	Cross-Cultural Adaptation
Arabic BCAM adaptation	Al-Khasawneh et al., 2016 [24]	–	α = 0.856–0.890 (+)	Inter-rater r = 0.97 (+)	–	Regression associations only (?)	r = 0.58, *p* < 0.01 (+)	–	Expert panel + cognitive interviews (+)
Arabic BCAM (APJCP module)	Alkhasawneh et al., 2016 [23]	–	α = 0.856–0.970 (+)	–	–	Demographic associations (?)	–	–	Arabic BCAM used; adaptation details limited (+/?)
Swahili BCAM	Wachira et al., 2017 [26]	EFA: loadings 0.52–0.84 (?)	α = 0.60–0.80 (+/?)	–	–	Partial hypothesis testing (?)	–	–	–
Persian BCAM	Heidari & Feizi, 2018 [27]	EFA + CFA: RMSEA = 0.046; CFI = 0.984 (+)	α = 0.882–0.919 (+)	ICC = 0.841; κ = 0.47–0.81 (+)	SEM = 0.85; SDC = 2.36 (+)	Convergent & discriminant (+)	AUC = 0.822 (+)	–	Forward–back + pilot (+)
Chinese BCAM (C-BCAM)	Liu et al., 2020 [28]	CFA: CFI = 0.94 (+)	α = 0.84–0.94 (+)	Test–retest r = 0.72 (+)	–	Theoretical alignment (+)	–	–	Translation + cognitive interviews (+)
Chinese BCAM (outcome use)	Liu et al., 2020 [29]	–	Reported (descriptive only; not evaluated)	–	–	Exploratory associations (?)	–	–	Used validated C-BCAM (no new adaptation)
BCAM (CR-UK, Pakistan)	Ullah et al., 2021 [31]	–	α = 0.964 (+)	–	–	Exploratory associations (?)	Content validity = 0.93 (+)	–	–
Turkish BCAM-Tr	Baş & Ursavaş, 2021 [33]	CFA: RMSEA = 0.078; CFI = 0.90 (+)	α = 0.89 (+)	ICC = 0.89 (+)	–	Known groups (+)	–	–	I-CVI = 1.00 (+)
Malay BCAM-M	Yusuf et al., 2022 [32]	–	α = 0.89 (+)	–	–	Pre–post hypothesis testing (+)	–	Pre–post change (+)	Forward–back + pilot (+)
Sudanese modified BCAM	Lawis & Sabir, 2023 [34]	–	α = 0.825 (+)	–	–	Demographic correlations (?)	–	–	Expert review + pilot (+)
Greek BCAM	Papasozomenou et al., 2024 [35]	–	α = 0.85–0.89 (+)	–	–	χ^2^ associations only (?)	–	–	Forward–back translation (+)

Note: COSMIN rating symbols: + = sufficient; - = insufficient; ? = indeterminate.

**Table 11:** Meta-analysis summary.

Measurement Property	Summarized Result	Heterogeneity/Model/Bias	Pooled N	Overall Rating	Quality of Evidence
Internal consistency (Cronbach’s α)	Pooled Cronbach’s α = 0.89 (95% CI 0.85–0.92)	Q(10) = 250.2, *p* < 0.001; τ^2^ = 0.055; I^2^ = 97.2%; random-effects model; trim-and-fill: 0 studies added	12,156 (11 studies)	? (consistently high α values; insufficient evidence for structural validity across versions)	Low

Footnotes: Meta-analytic reliability was synthesized using a random-effects model with pooled Cronbach’s α as the effect size. Heterogeneity statistics (Q, τ^2^, I^2^) reflect variation in internal consistency estimates between studies, not measurement error within studies. Funnel asymmetry and potential small-study bias were evaluated via trim-and-fill. “?” indicates that the overall COSMIN rating for internal consistency remains indeterminate due to the absence of structural validity in most versions. GRADE certainty reflects pooled synthesis (risk of bias, inconsistency, indirectness, and imprecision). Abbreviations (meta-analysis): Q = Cochran’s heterogeneity statistic; τ^2^ = between-study variance; I^2^ = proportion of variance attributable to heterogeneity; CI = confidence interval.

**Table 12:** Summary of certainty of evidence for Breast-CAM measurement properties using COSMIN-modified GRADE.

Measurement Property	Number of Studies	Summary of Findings	Certainty of Evidence (GRADE)	Reasons for Downgrading
Internal consistency	11	Pooled α = 0.89 (95% CI 0.85–0.92)	Low	Inconsistency; ceiling rule
Structural validity	3	Three studies evaluated dimensionality using factor analysis (EFA and/or CFA); most versions did not assess structural validity	Low	Risk of bias; inconsistency
Content validity	12	Seven sufficient; three indeterminate	Low	Indirectness; reporting gaps
Reliability (test–retest)	2	ICC > 0.70 in both	Very Low	Imprecision; inconsistency; limited sample
Measurement error	1	SEM/SDC reported; MIC absent	Very Low	Imprecision; indirectness
Construct validity	8	Four confirmed hypotheses; others exploratory	Low	Inconsistency; lack of prespecified hypotheses
Criterion validity	2	Two studies assessed validity using indirect comparators (no gold standard); inconsistent results	Very Low	Indirectness; risk of bias; imprecision
Responsiveness	4	Improvement following intervention	Low	Indirectness; absence of MIC
Cross-cultural adaptation	14	Seven complete adaptations; seven partial	Not graded	Not applicable (pre-psychometric process)

**Table 13:** Certainty summary per measurement property.

Measurement Property	Overall Rating (+/−/?)	Certainty of Evidence
Structural validity	−	Low
Internal consistency	?	Low
Content validity	+/?	Low
Reliability (test–retest)	?	Very low
Measurement error	−	Very low
Construct validity (hypothesis testing)	+/?	Low
Criterion validity	−	Very low
Responsiveness	+	Low
Cross-cultural adaptation	N/A	Not graded

Note: + = sufficient, − = insufficient, ? = indeterminate, N/A = not assessed as a psychometric property.

**Table 14:** Key priorities for strengthening the psychometric evaluation and application of the Breast-CAM.

Measurement Domain	Identified Gap in Current Evidence	Recommendation for Future Research
Cross-cultural adaptation	Several studies relied primarily on translation without full cultural validation procedures	Apply standardized cross-cultural adaptation procedures, including forward–backward translation, expert review, cognitive interviewing, and pilot testing
Structural validity	Factor structure rarely examined or inconsistently reported across studies	Conduct confirmatory factor analysis (CFA) or item response theory (IRT) analyses to establish the dimensional structure of the instrument
Internal consistency	Cronbach’s α frequently reported without prior confirmation of dimensionality	Evaluate internal consistency only after structural validity has been established and report reliability estimates separately for each subscale
Test–retest reliability	Temporal stability rarely assessed	Assess test–retest reliability using intraclass correlation coefficients (ICC) with clearly defined and appropriate retest intervals
Measurement error	Measurement error indices not reported in most studies	Report indices such as the standard error of measurement (SEM) and smallest detectable change (SDC)
Criterion validity	No established gold standard available for comparison	Explore associations with related outcomes such as screening participation, breast cancer knowledge scores, or other validated awareness measures
Responsiveness	Ability of the instrument to detect change after interventions rarely examined	Evaluate responsiveness in longitudinal studies and educational or awareness-raising interventions
Population diversity	Evidence mainly derived from limited geographical regions and populations	Validate the Breast-CAM in diverse populations, including rural communities, low-literacy groups, and underserved populations
Reporting quality	Inconsistent reporting of psychometric evaluation across studies	Follow COSMIN methodological standards and PRISMA reporting recommendations for future validation studies

## Data Availability

The original contributions presented in this study are included in the article and in the Supplementary Materials.

## Supplementary Materials

The following supporting information can be downloaded at: https://www.mdpi.com/article/10.3390/cancers18060956/s1, Supplementary File S1: PRISMA-COSMIN 2024 checklist for systematic reviews of outcome measurement instruments; Supplementary File S2: Search strategy; Supplementary File S3: COSMIN Risk of Bias and Measurement Properties Extraction Sheet; Supplementary Appendix S1: List of Excluded Full-Text Articles With Reasons; Supplementary Table S1: Study-level rating.

## Abbreviations

The following abbreviations are used in this manuscript:

Instrument and Framework	
ASReview	Active Learning for Systematic Reviews
BCAM	Breast Cancer Awareness Measure
Breast-CAM	Breast Cancer Awareness Measure
COSMIN	COnsensus-based Standards for the selection of health Measurement INstruments
GRADE	Grading of Recommendations Assessment, Development and Evaluation
OMI	Outcome Measurement Instrument
PRISMA	Preferred Reporting Items for Systematic Reviews and Meta-Analyses
PRISMA-S	PRISMA Extension for Reporting Literature Searches
PROSPERO	International Prospective Register of Systematic Reviews
Psychometrics and Statistics	
**α**	Cronbach’s Alpha
AUC	Area Under the Receiver Operating Characteristic Curve
CFA	Confirmatory Factor Analysis
CFI	Comparative Fit Index
CI	Confidence Interval
CVI	Content Validity Index
EFA	Exploratory Factor Analysis
ICC	Intraclass Correlation Coefficient
I^2^	I-squared Statistic
I-CVI	Item-level Content Validity Index
IQR	Interquartile Range
κ	Cohen’s Kappa
LoA	Limits of Agreement
MIC	Minimal Important Change
MID	Minimal Important Difference
Q	Cochran’s Heterogeneity Statistic
RMSEA	Root Mean Square Error of Approximation
ROC	Receiver Operating Characteristic
S-CVI	Scale-level Content Validity Index
SD	Standard Deviation
SDC	Smallest Detectable Change
SEM	Standard Error of Measurement
SRMR	Standardized Root Mean Square Residual
τ^2^	Between-study Variance
TLI	Tucker–Lewis Index
Study Design, Settings, and Organizations	
CRUK	Cancer Research UK
EU-CTR	EU Clinical Trials Register
FGDs	Focus Group Discussions
ICTRP	International Clinical Trials Registry Platform
INR	International Nursing Review
APJCP	Asian Pacific Journal of Cancer Prevention
NHS	National Health Service
OPD	Outpatient Department
PEP	Promoting Early Presentation
PHC	Primary Health Care Center
RCT	Randomized Controlled Trial
