# Supplementary material for: Psychometric Properties of the Breast Cancer Awareness Measure (Breast-CAM): A Systematic Review and Meta-Analysis
Source: Cancers (Basel). 2026 Mar 15;18(6):956. doi: 10.3390/cancers18060956 (PMC13025391; doi:10.3390/cancers18060956)
Supplement: Supplementary file 1 [file cancers-18-00956-s001.zip › Supplementary_Appendix_S1_List of Excluded Full-Text Articles With Reasons.pdf]

No.	Citation	Exclusion category	Detailed reason for exclusion
1	Abbas, M. O., & Baig, M. (2023). Knowledge and practice concerning breast cancer risk factors and screening among females in UAE. *Asian Pacific Journal of Cancer Prevention*, 24(2), 479–486. https://doi.org/10.31557/APJCP.2023.24.2.479	Awareness-only outcome	Cross-sectional KAP study; newly designed questionnaire (partially adapted BCAM) was used to describe awareness and screening practices only;
2	Abdou, A., Van Hal, G., & Dille, I. (2020). Validation of a BCAM (Breast Cancer Awareness Measure) tool for women and midwives in Niger. *Journal of Cancer Policy*, 24, 100253. https://doi.org/10.1016/j.jcpo.2020.100253	Mixed-gender population	Study included women, midwives and a mixed gender in group of specialists (not restricted to adult women as defined); psychometric properties reported in a broader, non-target population.
3	Abdou, A., Van Hal, G., & Dille, I. (2019). Awareness, attitudes and practices of women related to breast cancer in Niamey and Zinder. https://doi.org/10.21203/rs.2.17180/v1	Thesis / non–peer-reviewed	Report not published as a peer-reviewed journal article; descriptive awareness and practices only; no formal psychometric analysis of BCAM.
4	Abdou, A. (2022). *Breast cancer and breast cancer screening in Niger: An epidemiological and public health prospect* (Doctoral dissertation). https://repository.uantwerpen.be/docman/irua/3e48b1motoMb0	Thesis / dissertation / non–peer-reviewed	Doctoral thesis; not peer-reviewed journal research; BCAM not analysed as a stand-alone measurement instrument with formal psychometric evaluation.
5	Al Bashir, S., AlBarakat, M. M., Alabedalhalim, K. K., Al-Khalaileh, A., Alassaf, A., Saleh, O., … Alzoubi, K. H. (2024). Knowledge of cancer symptoms and risk factors: A cross-sectional study from a developing country. *Medicine*, 103(XX), e37823. https://doi.org/10.1097/MD.0000000000037823	Non-BCAM (generic CAM or other tool)	Used generic cancer awareness measures for multiple cancers in a mixed-gender sample; BCAM was not used and no psychometric analysis relevant to BCAM was reported.
6	Al-Hosni, K., Chan, M. F., & Al-Azri, M. (2023). Effectiveness of an educational program on awareness of breast cancer risk factors, symptoms, and barriers to seeking medical help among adolescent Omani school students—An interventional study. *Current Oncology*, 30(4), 314. https://doi.org/10.3390/curroncol30040314	Adolescent population	Intervention among school students (adolescents); breast cancer awareness only; no psychometric evaluation.
7	Al-Khamis, N. K. (2018). Low awareness of breast cancer and considerable barriers to early presentation among Saudi women at a primary care setting. *Journal of Cancer Education*, 33(2), 391–398. https://doi.org/10.1007/s13187-016-1119-x	Awareness-only outcome	Used a study-specific awareness/barriers questionnaire; no psychometric evaluation.
8	Al-Mousa, D. S., Alakhras, M., Hossain, S. Z., Al-Sa'di, A. G., Al Hasan, M., Al-Hayek, Y., & Brennan, P. C. (2020). Knowledge, attitude and practice around breast cancer and mammography screening among Jordanian women. *Breast Cancer: Targets and Therapy*, 12, 231–242. https://doi.org/10.2147/BCTT.S275445	Awareness-only outcome	KAP survey; focused on awareness and screening behaviour without psychometric evaluation.
9	Albinsaad, L., Alessa, M., Alraihan, J. I., Albesher, M. A., Alessa, H. A., & Almubarak, A. (2023). Women's knowledge and attitudes regarding the risk factors and warning signs of breast cancer in the Eastern Province, Saudi Arabia: A cross-sectional study. *Cureus*, 15(3), e49463. https://doi.org/10.7759/cureus.49463	Adolescent population	Conducted among younger/college-age women; awareness and attitudes only; no psychometric evaluation.
10	Aladien, Y. (2024). *Assessment of women's knowledge and awareness about breast cancer early detection and screening in women aged below 40 years in Palestine* (Doctoral dissertation).	Thesis / dissertation / non–peer-reviewed	Doctoral thesis in women <40 years; descriptive awareness only; no psychometric evaluation; not peer reviewed.
11	Alanzi, T. M., Alobrah, A., Alhumaidi, R., & Aloraifi, S. (2018). Evaluation of the Snapchat mobile social networking application for breast cancer awareness among Saudi students in the Dammam region of the Kingdom of Saudi Arabia. *Breast Cancer: Targets and Therapy*, 10, 113–118. https://doi.org/10.2147/BCTT.S166135	Adolescent population	Intervention among students via social media; assessed changes in awareness only; no psychometric evaluation.
12	Albanghali, M. A., Alnemari, R. K., Al Ghamdi, R. B., Gomaa, F. A. M., Alzahrani, T. A., Al Ghamdi, A. S., … Othman, B. A. (2025). Assessing breast cancer awareness among women in Al Baha, Saudi Arabia: A cross-sectional study using the Breast Cancer Awareness Measure (BCAM). *Medical Sciences*, 13(1), 24. https://doi.org/10.3390/medsci13010024	Awareness-only outcome	Used BCAM to describe awareness levels; no psychometric evaluation.
13	Albayrak, A., & Cengiz, K. N. (2024). Assessment of breast cancer awareness among female pharmacy students at a university in Turkey. *BMC Medical Education*, 24, 53. https://doi.org/10.1186/s12909-024-05353-x	Adolescent population	Young pharmacy-student sample; awareness only; no psychometric evaluation.
14	Alhumaid, A. A., Alshahrani, W., Al Qahtani, S. M., et al. (2024). Knowledge and level of awareness regarding breast cancer and practices of breast screening methods among female Riyadh citizens. *Cureus*, 16(5), e59996. https://doi.org/10.7759/cureus.59996	Awareness-only outcome	Cross-sectional community KAP survey; awareness/screening practice only, no psychometric evaluation.
15	Alizadeh Sabeg, P., Mehrabi, E., Nourizadeh, R., Poursharifi, H., & Mousavi, S. (2019). The effect of counseling on breast cancer awareness in rural Iranian women: A randomized controlled clinical trial. *Journal of Cancer Education*, 34(6), 1080–1088. https://doi.org/10.1007/s13187-018-1411-z	Awareness-only outcome	Counselling intervention evaluated awareness changes only; no psychometric evaluation.

**16**	Almajed, H. (2023). *Breast cancer awareness among female residents of Kuwait* (Doctoral dissertation).	Thesis / dissertation / non–peer-reviewed	Doctoral thesis; descriptive awareness only; BCAM psychometric properties not reported.
**17**	Alsaraireh, A., & Darawad, M. W. (2018). Breast cancer awareness, attitude and practices among female university students: A descriptive study from Jordan. *Health Care for Women International*, 39(5), 571–583. https://doi.org/10.1080/07399332.2017.1368516	Adolescent population	University-student population; descriptive awareness/attitude/practice outcomes only; no psychometric analysis.
**18**	Alshafie, M., Bitar, A., Alameer, M. B., et al. (2025). *Female healthcare undergraduates' scientific readiness for raising breast cancer awareness in a Syrian conservative community. BMC Medical Education, 25*(111). https://doi.org/10.1186/s12909-024-06586-6	Awareness-only outcome	Applied Arabic BCAM for knowledge assessment only; no psychometric evaluation.
**19**	Baburajan, C., Pushparani, M. S., Lawenya, M., Lukose, L., & Johnson, A. R. (2022). Are rural women aware of breast cancer and do they practice breast self-examination? A cross-sectional study in a rural hospital in South India. *Indian Journal of Cancer*, 59(1), 88–93. https://doi.org/10.4103/ijc.IJC_799_19	Awareness-only outcome	Assessed awareness and BSE practice; no psychometric evaluation.
**20**	Bajaj, K., Ravi, A., Thakur, U., Mishra, A., & Khare, S. (2021). Awareness about breast cancer in first-year junior residents at a tertiary care institute in India: A cross-sectional study. *Medical Journal Armed Forces India*, 77(Suppl 1), S76–S82. https://doi.org/10.1016/j.mjafi.2020.12.006	Adolescent population	Young/junior doctors; awareness only; no psychometric evaluation.
**21**	Bao, H., Liu, L., Cong, S., Wang, F., Yu, L., Fang, L., Duan, X., Tan, F., Yu, Z., & Wang, L. (2023). Breast Cancer Awareness and Association with Frequency of Screening Among Women - China, 2020. *China CDC weekly*, *5*(15), 327–332. https://doi.org/10.46234/ccdcw2023.063	Awareness-only outcome	National survey linking awareness to screening frequency; no psychometric assessment.
**22**	Busakhala, N. W., Chite, F. A., Wachira, J., Naanyu, V., Kisuya, J. W., Keter, A., … Inui, T. (2016). Screening by clinical breast examination in Western Kenya: Who comes? *Journal of Global Oncology*, 2(6), 398–406. https://doi.org/10.1200/JGO.2015.000687	Mixed-gender population	Study of attendance for clinical breast examination; included both women and men, no psychometric evaluation.
**23**	Campbell, J., Pyer, M., Rogers, S., Jones, J., Ramirez, A. J., & Forbes, L. J. L. (2016). Promoting early presentation of breast cancer in women over 70 years old in general practice. *Journal of Public Health*, 38(4), 778–785. https://doi.org/10.1093/pubmed/fdv125	Awareness-only outcome	Intervention trial; no psychometrics evaluation
**24**	Chao, C. A., Huang, L., Visvanathan, K., Mwakatobe, K., Masalu, N., & Rositch, A. F. (2020). Understanding women's perspectives on breast cancer is essential for cancer control: Knowledge, risk awareness, and care-seeking in Mwanza, Tanzania. *BMC Public Health*, 20, 1027. https://doi.org/10.1186/s12889-020-09010-y	Awareness-only outcome	Focus on knowledge and care-seeking; no psychometric evaluation.
**25**	Colman, R., Kirkman, A., Jackson, J., Golbourn, L., Ozaki, A., & Kotera, Y. (2023). Breast Cancer Understanding Among University Students: A Rapid Review of Cross-country Comparisons: Student BC understanding: rapid review. *Archives of Breast Cancer*, *10*(2), 114–123. https://doi.org/10.32768/abc.2023102114-123	Review	Rapid review.
**26**	Dabbour, R., Khleifat, A., Tawalbeh, D., & Al Tete, R. (2022). Breast cancer knowledge among university female students in Jordan: A cross-sectional study. *HIV Nursing*, 22(2), 1–9.	Adolescent population	University students; awareness only; non-BCAM questionnaire; no psychometric analysis.
**27**	Das, S. (2022). *Breast cancer knowledge, attitude, and screening practices in African American college students* (Doctoral dissertation). Breast Cancer Knowledge, Attitude, and Screening Practices in African American College Students - ProQuest	Thesis / dissertation / non–peer-reviewed	College-student thesis; awareness and practices only; BCAM not used; no psychometric evaluation.
**28**	Dembinsky, M. (n.d.). *Breast awareness among black women in East London 2013–2015.* Breast awareness among black women in East London 2013-2015 - ReShare	Data archive / non-original research	Data archive entry; no accompanying peer-reviewed report with BCAM psychometrics.
**29**	Dodd, R. H., Forster, A. S., Sellars, S., Patnick, J., Ramirez, A. J., & Forbes, L. J. L. (2017). Promoting early presentation of breast cancer in older women: Sustained effect of an intervention to promote breast cancer awareness in routine clinical practice. *BMC Health Services Research*, 17, 706. https://doi.org/10.1186/s12913-017-2335-8	Awareness-only outcome	No psychometric evaluation.
**30**	Duong, L. T., Chen, H.-M., Liu, C.-Y., & Chiou, P.-Y. (2020). Factors affecting mammography screening behaviour among rural Vietnamese women. *European Journal of Cancer Care*, 29(6), e13300. https://doi.org/10.1111/ecc.13300	Awareness-only outcome	Focus on screening behaviour and predictors and awareness measures; no psychometric evaluation.
**31**	El Maouchi, P., Fakhreddine, O., Shmoury, A. H., El Zoghbi, M., Chamseddine, N., Abou Zeidane, R., … Assi, H. I. (2023). Breast cancer knowledge in Lebanese females with positive family history. *Medicine*, 102(XX), e32973. https://doi.org/10.1097/MD.0000000000032973	Awareness-only outcome	Assessed knowledge in high-risk women; no psychometric evaluation.
**32**	El-Shinawi, M., Youssef, A., Alsara, M., Aly, M. K., Mostafa, M., Yehia, A., … Mohamed, M. M. (2013). Assessing the level of breast cancer awareness among recently diagnosed patients in Ain Shams University Hospital. *Breast*, 22(6), 1210–1214. https://doi.org/10.1016/j.breast.2013.08.010	Awareness-only outcome	Awareness in recently diagnosed patients; no psychometric evaluation.
**33**	Elobaid, Y. E., Aw, T. C., Grivna, M., & Nagelkerke, N. (2014). Breast cancer screening awareness, knowledge, and practice among Arab	Awareness-only	KAP survey; no psychometric evaluation.

	women in the United Arab Emirates: A cross-sectional survey. *PLOS ONE*, 9(9), e105783. https://doi.org/10.1371/journal.pone.0105783	outcome	
**34**	Elshami, M., Abu Kmeil, H., Abu-Jazar, M., Mahfouz, I., Ashour, D., Aljamal, A., … Böttcher, B. (2018). Breast cancer awareness and barriers to early presentation in the Gaza-Strip: A cross-sectional study. *Journal of Global Oncology*, 4, 1–10. https://doi.org/10.1200/JGO.18.00095	Adolescent population	Sample based on young/college-age participants; awareness and barriers only; no psychometric evaluation.
**35**	Elshami, M., Al-Slaibi, I., Ghithan, R.J. *et al.* Women's awareness of breast cancer symptoms: a national cross-sectional study from Palestine. *BMC Public Health* **22**, 801 (2022). https://doi.org/10.1186/s12889-022-13224-7	Awareness-only outcome	Symptom awareness only; no psychometric evaluation.
**36**	Forbes, L. J. L., Atkins, L., Jupp, D., Green, D., Scanlon, K., & Ramirez, A. (2011). Evaluation of an intervention to promote breast cancer awareness and early presentation: The train-the-trainers breast health promotion course. *Psycho-Oncology*, 21(1), 144–151. https://doi.org/10.1002/pon.2078	Awareness-only outcome	BCAM-based awareness items used to evaluate training; no psychometric evaluation.
**37**	Forbes, L. J. L., Atkins, L., Thurnham, A., Layburn, J., Haste, F., & Ramirez, A. J. (2011). Breast cancer awareness and barriers to symptomatic presentation among women from different ethnic groups in East London. *British Journal of Cancer*, 105(10), 1474–1479. https://doi.org/10.1038/bjc.2011.406	Awareness-only outcome	Early work underpinning BCAM; awareness and barriers described, no psychometric evaluation.
**38**	Forster, A. S., Forbes, L. J. L., Abraham, C., Warburton, F. G., Douglas, E., & Ramirez, A.-J. (2014). Promoting early presentation of breast cancer: A preliminary evaluation of a written intervention. *Chronic Illness*, 10(1), 3–18. https://doi.org/10.1177/1742395313484071	Awareness-only outcome	Written intervention study: BCAM outcomes reported, no psychometric evaluation.
**39**	Ghoneim, A. A., Abdelmaksoud, B. A., Alruwaily, F., & Elsharkawy, N. B. (2023). Measurement of the extent of awareness of Al Jouf region residents with the importance and methods of breast cancer early detection in females. *Asian Pacific Journal of Cancer Prevention*, 24(3), 945–952. https://doi.org/10.31557/APJCP.2023.24.3.945	Mixed-gender population	Mixed-gender community sample; awareness-only.
**40**	Githaiga, J. N., Walter, F. M., Scott, S. E., Mwaka, A. D., & Moodley, J. (2019). Symptom awareness measures for breast and cervical cancer in sub-Saharan Africa: A scoping review. *South African Journal of Oncology*, 3, a78. https://doi.org/10.4102/sajo.v3i0.78	Scoping review	The study does not present original BCAM data or psychometric evaluation.
**41**	Haji Zaini, N., Haji Abdul Razak, N., Abdul Rahman, H., & Abdul-Mumin, K. H. (2022). Breast cancer knowledge and screening awareness among women attending well women clinic. *Indian Journal of Gynecologic Oncology*, 20(1), 5. https://doi.org/10.1007/s40944-021-00593-x	Awareness-only outcome	Clinic-based awareness and screening survey; no psychometric evaluation.
**42**	Hajian-Tilaki, K., Nikpour, M. Accuracy of self-perceived risk perception of breast cancer development in Iranian women. *BMC Women's Health* **21**, 93 (2021). https://doi.org/10.1186/s12905-021-01238-z	Awareness-only outcome	Focused on risk perception and accuracy; no psychometric evaluation.
**43**	Hamed, E., Alemrayat, B., Syed, M. A., Daher-Nashif, S., Rasheed, H. M. A., & Kane, T. (2022). Breast cancer knowledge, attitudes and practices amongst women in Qatar. *International Journal of Environmental Research and Public Health*, 19(7), 3995. https://doi.org/10.3390/ijerph19073995	Awareness-only outcome	KAP survey; no psychometric evaluation.
**44**	Hutajulu, S. H., Prabandari, Y. S., Bintoro, B. S., Wiranata, J. A., Widiastuti, M., Suryani, N. D., … Allsop, M. J. (2022). Delays in the presentation and diagnosis of women with breast cancer in Yogyakarta, Indonesia: A retrospective observational study. *PLOS ONE*, 17(2), e0262468. https://doi.org/10.1371/journal.pone.0262468	Awareness-only outcome	Focus on diagnostic delay; no psychometric evaluation.
**45**	Hussain, I., Majeed, A., Masood, I., Ashraf, W., Imran, I., Saeed, H., … Saleem, F. (2022). A national survey to assess breast cancer awareness among the female university students of Pakistan. *PLOS ONE*, 17(1), e0262030. https://doi.org/10.1371/journal.pone.0262030	Adolescent population	University students; awareness-only; no psychometric evaluation.
**46**	Ismail, H., Shibani, M., Zahrawi, H. W., et al. (2021). Knowledge of breast cancer among medical students in Syrian Private University, Syria: A cross-sectional study. *BMC Medical Education, 21*, 499. https://doi.org/10.1186/s12909-021-02673-0	Mixed gender population	Mixed-gender medical-student sample; knowledge assessment only.
**47**	Jomaa, M. K., Gado, N. M., Elgazawy, H., Sayed, F., Mousselhy, A., & Alfaar, A. S. (2015). Assessment of breast cancer screening awareness among relatives of Egyptian breast cancer patients. *Annals of Oncology*, 26(Suppl 4), iv120. https://doi.org/10.1093/annonc/mdv519.35	Awareness-only outcome	Screening awareness among relatives; no psychometric evaluation.
**48**	Kharaba, Z., Buabeid, M. A., Ramadan, A., Ghemrawi, R., Al-Azayzih, A., Al Meslamani, A. Z., & Alfoteih, Y. (2021). Knowledge, attitudes, and practices concerning breast cancer and self-examination among females in UAE. *Journal of Community Health*, 46(4), 601–609. https://doi.org/10.1007/s10900-021-00969-2	Awareness-only outcome	KAP of breast cancer and BSE; no psychometric evaluation.
**49**	Kizilkaya, M. C., Kilic, S. S., Bozkurt, M. A., Sibic, O., Ohri, N., Faggen, M., … Sayan, M. (2022). Breast cancer awareness among Afghan refugee women in Turkey. *eClinicalMedicine*, 53, 101459. https://doi.org/10.1016/j.eclinm.2022.101459	Awareness-only outcome	BCAM used to assess awareness, no psychometric evaluation.
**50**	Kizilkaya, M. C., Kilic, S., Dagistanli, S., Eren, M. F., Basaran, C., Ohri, N., & Sayan, M. (2023). Effectiveness of a telehealth patient education	Awareness-only	Telehealth intervention using BCAM outcomes; no psychometric

	intervention for breast cancer awareness and screening uptake among Afghan refugee women: A cross-sectional survey and feasibility study. *eClinicalMedicine*, 60, 102094. https://doi.org/10.1016/j.eclinm.2023.102094	outcome	evaluation.
**51**	Kuru Alici, N., Arikan Dönmez, A., Aktaş, O., & Zeytun, Z. (2025). The effect of simulation-based breast health education on breast cancer awareness and breast self-examination skills of Afghan refugee women: A randomized controlled trial. *BMC Public Health*, 25(1), 2048. https://doi.org/10.1186/s12889-025-23313-y	Awareness-only outcome	RCT used BCAM-type awareness scores to evaluate intervention; no psychometric evaluation.
**52**	Liu, N., Wang, J., Chen, D.-D., Sun, W.-J., & Zhang, W. (2019). Tools for the assessment of breast cancer screening beliefs in women: A literature review. *Journal of Comparative Effectiveness Research*, 8(11), 879–892. https://doi.org/10.2217/cer-2018-0142	Review / scoping / rapid review	Literature review of tools.
**53**	Lounis, M., Belkessa, S., Abdelhadi, S. *et al.* Breast cancer knowledge and practices amongst women in Algeria. *J Cancer Res Clin Oncol* **149**, 8843–8852 (2023). https://doi.org/10.1007/s00432-023-04786-z	Awareness-only outcome	Awareness and practices only; no psychometric evaluation.
**54**	Maureen, K. J., Magutah, K., Mogere, D. M., Kariuki, J., Willy, K., Muriira, M. A., & Chege, H. (2024). Knowledge, attitude and practices around breast cancer and screening services among women of reproductive age in Turbo sub-county, Kenya. *Heliyon*, *10*(11). doi: 10.1016/j.heliyon.2024.e31597	Awareness-only outcome	KAP cross-sectional survey; no psychometric evaluation.
**55**	Mardela, A. P., Maneewat, K., & Sangchan, H. (2017). Breast cancer awareness among Indonesian women at moderate-to-high risk. *Nursing & Health Sciences*, 19(3), 301–307. https://doi.org/10.1111/nhs.12345	Awareness-only outcome	High-risk women's awareness study; no psychometric evaluation.
**56**	Mehmood, Y. (2025). Perception and knowledge of breast cancer: Evaluating educational gaps among Saudi Arabian female medical students. *Anaesthesia, Pain & Intensive Care*, 29(2), 294–299. https://doi.org/10.35975/apic.v29i2.2718	Adolescent population	Female medical students; awareness/perception only; no psychometric analysis.
**57**	Miskeen, E., & Al-Shahrani, A. M. (2023). Breast cancer awareness among medical students, University of Bisha, Saudi Arabia. *Breast Cancer: Targets and Therapy*, 15, 131–141. https://doi.org/10.2147/BCTT.S403803	Mixed-gender population	Included both male and female medical students; awareness-only outcomes; no psychometric evaluation.
**58**	Moodley, J., Constant, D., Mwaka, A. D., Scott, S. E., & Walter, F. M. (2020). Mapping awareness of breast and cervical cancer risk factors, symptoms and lay beliefs in Uganda and South Africa. *PLOS ONE*, 15(10), e0240788. https://doi.org/10.1371/journal.pone.0240788	Awareness-only outcome	Mapping awareness and beliefs; no psychometric evaluation.
**59**	Naanyu, V., Asirwa, C. F., Wachira, J., Busakhala, N., Kisuya, J., Otieno, G., … Inui, T. (2015). Lay perceptions of breast cancer in Western Kenya. *World Journal of Clinical Oncology*, 6(5), 147–155. https://doi.org/10.5306/wjco.v6.i5.147	Awareness-only outcome	Qualitative/perception focus; no psychometric evaluation.
**60**	Nageeti, T. H., Abdelhameed, A. A. N., Jastania, R. A., & Felemban, R. M. (2017). Perspective of Saudi women in the Makkah region on breast cancer awareness. *Journal of Family & Community Medicine*, 24(2), 94–101. https://doi.org/10.4103/2230-8229.205116	Awareness-only outcome	Awareness and perspectives; no psychometric evaluation.
**61**	Newton, M. V., & Palanivelrajan, V. V. (2024). How "breast aware" are the Indian women? A study among the women visiting a tertiary care, referral, and teaching hospital. *Annals of African Medicine*, 23(1), 1–7. https://doi.org/10.4103/aam.aam_194_23	Awareness-only outcome	Clinic-based awareness measure; no psychometric evaluation.
**62**	Ngan, T. T., Jenkins, C., Van Minh, H., Donnelly, M., & O'Neill, C. (2022). Breast cancer screening practices among Vietnamese women and factors associated with clinical breast examination uptake. *PLOS ONE*, 17(6), e0269228. https://doi.org/10.1371/journal.pone.0269228	Awareness-only outcome	Screening practices and associated factors; awareness; no psychometric evaluation.
**63**	Niksic, M., Rachet, B., Duffy, S. W., Quaresma, M., Møller, H., & Forbes, L. J. L. (2016). Is cancer survival associated with cancer symptom awareness and barriers to seeking medical help in England? An ecological study. *British Journal of Cancer*, 115(7), 876–886. https://doi.org/10.1038/bjc.2016.246	Non-BCAM (generic CAM)	Used generic Cancer Awareness Measures (CAM) across cancers; mixed-gender ecological analysis; not BCAM-specific.
**64**	Niksic, M., Rachet, B., Warburton, F. G., & Forbes, L. J. L. (2016). Ethnic differences in cancer symptom awareness and barriers to seeking medical help in England. *British Journal of Cancer*, 115(1), 136–144. https://doi.org/10.1038/bjc.2016.158	Non-BCAM (generic CAM)	Used generic CAM in mixed-gender population; not breast-specific.
**65**	Niksic, M., Rachet, B., Warburton, F. G., Wardle, J., Ramirez, A. J., & Forbes, L. J. L. (2015). Cancer symptom awareness and barriers to symptomatic presentation in England – Are we clear on cancer? *British Journal of Cancer*, 113(3), 533–542. https://doi.org/10.1038/bjc.2015.164	Non-BCAM (generic CAM))	Clear on Cancer campaign paper; used generic CAM; not BCAM-specific.
**66**	Omar, A., Bakr, A., & Ibrahim, N. (2020). Female medical students' awareness, attitudes, and knowledge about early detection of breast cancer in Syrian Private University, Syria. *Heliyon*, 6, e03819. https://doi.org/10.1016/j.heliyon.2020.e03819	Adolescent population	Medical student sample; awareness-only outcomes; no psychometric evaluation.
**67**	Peniamina, R., McNoe, B., & Signal, L. (2023). Public awareness of cancer risk factors & support for prevention policies in Aotearoa New Zealand: A focus on alcohol and diet. Public awareness of cancer risk factors & support for prevention policies in Aotearoa New Zealand: A focus on alcohol and diet - University of Otago	Non-BCAM (generic CAM)	Public awareness report across cancers and sexes; tool not BCAM.

68	Potluri, T. S., Vadlamani, S., Gujjarlapudi, C., Nerusu, N. G., & Rongala, M. V. (2023). An educational intervention study to enhance breast cancer awareness among women and primary healthcare providers of an urban health center area, Visakhapatnam. *Journal of Family Medicine and Primary Care*, 12(10), 1850–1858. https://doi.org/10.4103/jfmpc.jfmpc_932_23	Awareness-only outcome	Educational intervention: awareness measured; no psychometric evaluation.
69	Qasim, S., Tayyab, H., Zulqadar, K., Masood, S., Qasim, T. B., Zubair, Z., … Qasim, T. B. (2020). Breast cancer knowledge and perceived barriers to help seeking among pre-clinical and clinical female medical students of King Edward Medical University, Lahore: A cross-sectional study. *BMC Medical Education*, 20, 294. https://doi.org/10.1186/s12909-020-02132-2	Adolescent population	Female medical students; knowledge/barriers only; no psychometric evaluation.
70	Qedair, J. T., Al Qurashi, A. A., Alfayea, T., Mortada, H., Alsudais, A., Almuntashiri, S., … Hakami, A. Y. (2022). Level and predictors of breast cancer awareness among Saudi women: A nationwide study. *Women's Health*, 18, 17455057221133835. https://doi.org/10.1177/17455057221133835	Awareness-only outcome	Nationwide awareness predictors; no psychometric evaluation.
71	Radi, S. M. (2013). Breast cancer awareness among Saudi females in Jeddah. *Asian Pacific Journal of Cancer Prevention*, 14(7), 4307–4312. https://doi.org/10.7314/APJCP.2013.14.7.4307	Awareness-only outcome	Awareness-only survey; no psychometric evaluation.
72	Rahman, S. A. A., Kherbek, H., Ismail, S., Rahman, A. A., Zahlout, J., Abboud, I., ... & Alshehabi, Z. (2023). Breast cancer awareness among women in the Syrian Coast: a cross-sectional study. *Annals of medicine and surgery*, *85*(6), 2474-2479. doi: 10.1097/MS9.0000000000000753	Awareness-only outcome	Cross-sectional awareness; no psychometric evaluation.
73	Rassool, M. Y. A., Soodeen-Lalloo, A. K., & Sadally, S. B. (2010). Evaluating breast cancer awareness of Mauritian women (>20 years) using the UK Breast Cancer Awareness Measure. Evaluating-Breast-Cancer-awareness-of-Mauritian-Women-20-years-using-the-UK-Breast-Cancer-Awareness-measure.pdf	Poster / non–peer-reviewed	Conference poster; not a full peer-reviewed article; BCAM results reported in brief with no psychometric evaluation.
74	Reidy, M., Denieffe, S., & Foran, S. (2018). Exploring breast cancer and screening awareness among Irish women with intellectual disabilities. *British Journal of Learning Disabilities*, 46(3), 193–201. https://doi.org/10.1111/bld.12225	Awareness-only outcome	Special population (intellectual disabilities); exploratory awareness only; no psychometric evaluation.
75	Redha, A. N. (2015). *Immigrant Arab Women: Knowledge, Beliefs and Attitudes towards Breast Cancer and Cancer Awareness Practice* (Doctoral dissertation, University of Portsmouth). Final_Version_of_the_Thesis_Afrah_Redha.pdf	Thesis / dissertation / non–peer-reviewed	Doctoral dissertation; awareness, beliefs and practices only; no psychometric evaluation.
76	Saeed, R., Usmani, M., Durrani, N., Javaid, H., & Tahir, H. (2025). Knowledge, perception and utilization of breast cancer screening among women visiting primary care clinics. *Journal of Family Medicine and Primary Care*, 14(2), 637–642. https://doi.org/10.4103/jfmpc.jfmpc_1044_24	Awareness-only outcome	Knowledge/perception/screening, no psychometric evaluation.
77	Sana, M. M., Radhakrishnan, R., Harikrishna, S., et al. (2025). Evaluating breast cancer awareness and self-examination practices among women in low socio-economic areas of South India. *Indian Journal of Gynecologic Oncology*, 23, 42. https://doi.org/10.1007/s40944-025-00972-8	Awareness-only outcome	Evaluated awareness and BSE practice; no psychometric evaluation.
78	Schliemann, D., Htay, M. N. N., Dahlui, M., Paramasivam, D., Cardwell, C. R., Ibrahim Tamin, N. S. B., … Donnelly, M. (2020). Impact of a mass media campaign on breast cancer symptoms awareness and screening uptake in Malaysia: Findings from a quasi-experimental study. *BMJ Open*, 10(5), e036503. https://doi.org/10.1136/bmjopen-2019-036503	Awareness-only outcome	Mass media campaign evaluation; no psychometric evaluation.
79	Shakor, J. K., Mohammed, A. K., & Karotia, D. (2019). Determinants of breast self-examination practice amongst Iraqi/Sulaimani Women using Champion Health belief model and breast CAM. *International Journal of Medical Research & Health Sciences*, *8*(9), 51-59.	Awareness-only outcome	Used newly designed questionnaires (partially adapted BCAM and combined with HBM constructs; no psychometric evaluation.
80	Shoukat, Z., & Shah, A. J. (2023). Breast cancer awareness and associated factors among women in Pakistan: A cross-sectional descriptive study. *Asian Pacific Journal of Cancer Prevention*, 24(5), 1561–1561. https://doi.org/10.31557/APJCP.2023.24.5.1561	Awareness-only outcome	Awareness and associated factors only; no psychometric evaluation.
81	Solanki A., Tulsi A. (2016). Assessment of level of breast cancer awareness among women registered at a regional cancer centre, 38th Annual Conference of the Association of Radiation Oncologists of India, AROICON 2016. Journal of Cancer Research and Therapeutics.	Awareness-only outcome	Awareness-only; no psychometric evaluation.
82	Sujha Subramanian et al. Establishing Cohorts to Generate the Evidence Base to Reduce the Burden of Breast Cancer in Sub-Saharan Africa: Results From a Feasibility Study in Kenya. *JGO* **5**, 1-10(2019). doi:10.1200/JGO.18.00225	Awareness-only outcome	Cohort feasibility; no psychometric evaluation.
83	Yang, S., Li, P., Yu, L., Liu, N., Wang, J., Guo, P., … Zhang, W. (2022). Breast cancer awareness based on health information literacy and influential factors among female nursing students in China. *Journal of Cancer Education*, 37(6), 1594–1602. https://doi.org/10.1007/s13187-020-01844-9	Adolescent population	Nursing students; awareness and health information literacy only; no psychometric evaluation.
84	Yadav, P., & Jaroli, D. P. (2010). Breast cancer: Awareness and risk	Adolescent	College-going younger women;

	factors in college-going younger age group women in Rajasthan. *Asian Pac J Cancer Prev*, *11*(2), 319-22.	population	awareness-only; no psychometric evaluation.
**85**	Yasin, G., & Subke, A. A. (2024). Breast cancer screening awareness and associated factors among Saudi females: A cross-sectional study in Jeddah, Saudi Arabia (2024). *Cureus*, 16(2), e60337. https://doi.org/10.7759/cureus.60337	Awareness-only outcome	Screening awareness and associated factors; no psychometric evaluation.
**86**	Yeung, M. P. S., Chan, E. Y. Y., Wong, S. Y. S., Yip, B. H. K., & Cheung, P. S.-Y. (2019). Hong Kong females' breast cancer awareness measure: Cross-sectional survey. *World Journal of Clinical Oncology*, 10(2), 98–109. https://doi.org/10.5306/wjco.v10.i2.98	Awareness-only outcome	The study describes awareness; no psychometric evaluation.
**87**	Lim, P. Y., & Yusuf, A. (2022). Knowledge of Breast Cancer Among Urban Women in the South of Peninsular Malaysia. *Malaysian Journal of Medicine and Health Sciences*, *18*(6), 27–34. https://doi.org/10.47836/mjmhs18.6.5	Awareness-only outcome	Knowledge-only survey; no psychometric evaluation.
**88**	Zhu, L., Zhou, Q., Huang, Z., Yang, Y., Du, Y., Zhao, Y., … Chen, W. (2024). Factors influencing breast cancer awareness in rural Southwest China: A cross-sectional study. *International Journal of Women's Health*, 16, 23–35. https://doi.org/10.2147/IJWH.S453857	Awareness-only outcome	Assessed factors influencing awareness; no psychometric evaluation.
