# Supplementary material for: Psychometric Properties of the Breast Cancer Awareness Measure (Breast-CAM): A Systematic Review and Meta-Analysis
Source: Cancers (Basel). 2026 Mar 15;18(6):956. doi: 10.3390/cancers18060956 (PMC13025391; doi:10.3390/cancers18060956)
Supplement: Supplementary file 1 [file cancers-18-00956-s001.zip › Supplementary_File_S1_PRISMA-COSMIN+Reporting+checklist+Full+reports+-+version+January+2024.pdf]

PRISMA - COSMIN OUTCOME Measurement Instruments

Full reports

Section and Topic	#	Checklist item[a]	Location
TITLE			
Title	1	Identify the report as a systematic review and include as applicable the following (in any order): outcome domain of interest, population<br>of interest, name/type of OMIs of interest, and measurement properties of interest.	p. 1
ABSTRACT			
OPEN SCIENCE			
Funding[b]	2.2	Specify the primary source of funding for the review.	p.41
Registration	2.3	Provide the register name and registration number.	p. 4
BACKGROUND			
Objectives	2.4	Provide an explicit statement of the main objective(s) or question(s) the review addresses.	p. 1
METHODS			
Eligibility criteria	2.5	Specify the inclusion and exclusion criteria for the review.	p. 4
Information sources	2.6	Specify the information sources (e.g., databases, registers) used to identify studies and the date when each was last searched.	pp. 4-6
Risk of bias	2.7	Specify the methods used to assess risk of bias in the included studies.	pp. 8-10
Measurement properties	2.8	Specify the methods used to rate the results of a measurement property.	pp. 8-10
Synthesis methods	2.9	Specify the methods used to present and synthesize results.	pp. 10-12
RESULTS			
Included studies	2.10	Give the total number of included OMIs and study reports.	pp. 12-27
Synthesis of results	2.11	Present the syntheses of results of OMIs, indicating the certainty of the evidence.	pp. 27-36
DISCUSSION			
Limitations of evidence	2.12	Provide a brief summary of the limitations of the evidence included in the review (e.g., study risk of bias, inconsistency, and imprecision).	pp. 37-38
Interpretation	2.13	Provide a general interpretation of the results and important implications.	pp. 38-40
PLAIN LANGUAGE SUMMARY			
Plain language summary	3	If allowed by the journal, provide a plain language summary with background information and key findings.	p.1 Addressed in Simple Summary
OPEN SCIENCE			

PRISMA - COSMIN OUTCOME Measurement Instruments

Full reports

Section and Topic	#	Checklist item[a]	Location
Registration and protocol	4a	Provide registration information for the review, including register name and registration number, or state that the review was not registered.	p. 4
	4b	Indicate where the review protocol can be accessed, or state that a protocol was not prepared.	p.4 PROSPERO (CRD420251158142); protocol available via PROSPERO
	4c	Describe and explain any amendments to information provided at registration or in the protocol.	Not applicable; no amendments to the registered protocol were made.
Support	5	Describe sources of financial or non-financial support for the review, and the role of the funders in the review.	p. 41, Funding
Competing interests	6	Declare any competing interests of review authors.	p. 41
Availability of data, code, and other materials	7	Report which of the following are publicly available and where they can be found: template data collection forms; data extracted from included studies; data used for all analyses; analytic code; any other materials used in the review.	p. 41 Data availability statement
INTRODUCTION			
Rationale	8	Describe the rationale for the review in the context of existing knowledge.	p. 3 Introduction
Objectives	9	Provide an explicit statement of the objective(s) or question(s) the review addresses and include as applicable the following (in any order): outcome domain of interest, population of interest, name/type of OMIs of interest, and measurement properties of interest.	p. 3 Introduction
METHODS			
Followed guidelines	10	Specify, with references, the methodology and/or guidelines used to conduct the systematic review.	pp.3-4 Materials and Methodes
Eligibility criteria	11	Specify the inclusion and exclusion criteria for the review.	p. 4 Eligibility criteria
Information sources	12	Specify all databases, registers, preprint servers, websites, organizations, reference lists and other sources searched or consulted to identify studies. Specify the date when each source was last searched or consulted.	pp. 4-6 Information sources
Search strategy	13	Present the full search strategies for all databases, registers, and websites, including any filters and limits used.	p. 6 Search strategy
Selection process	14	Specify the methods used to decide whether a study met the inclusion criteria of the review, e.g., including how many reviewers screened each record and each report retrieved, whether they worked independently, and if applicable, details of automation tools/AI used in the process.	p. 6 Selection process

PRISMA - COSMIN OUTCOME Measurement Instruments

Full reports

Section and Topic	#	Checklist item[a]	Location
Data collection process	15	Specify the methods used to collect data from reports, e.g., including how many reviewers collected data from each report, whether they worked independently, any processes for obtaining or confirming data from study investigators, and if applicable, details of automation tools/AI used in the process.	p.7 Data collection process
Data items	16	List and define which data were extracted (e.g., characteristics of study populations and OMIs, measurement properties' results, and aspects of feasibility and interpretability). Describe methods used to deal with any missing or unclear information.	p. 7 Data items
Study risk of bias assessment	17	Specify the methods used to assess risk of bias in the included studies, e.g., including details of the tool(s) used, how many reviewers assessed each study and whether they worked independently, and if applicable, details of automation tools/AI used in the process.	p. 8 Study risk of bias assessment
Measurement properties	18	Specify the methods used to rate the results of a measurement property for each individual study and for the summarized or pooled results, e.g., including how many reviewers rated each study and whether they worked independently.	pp. 8-10 Measurement properties
Synthesis methods	19a	Describe the processes used to decide which studies were eligible for each synthesis.	pp. 10-11
	19b	Describe any methods used to synthesize results.	p. 11
	19c	If applicable, describe any methods used to explore possible causes of inconsistency among study results (e.g., subgroup analysis).	p. 11
	19d	If applicable, describe any sensitivity analyses conducted to assess robustness of the synthesized results.	p. 11
Certainty assessment	20	Describe any methods used to assess certainty (or confidence) in the body of evidence.	pp. 11-12
Formulating recommendations	21	If appropriate, describe any methods used to formulate recommendations regarding the suitability of OMIs for a particular use.	p. 12
RESULTS			
Study selection	22a	Describe the results of the search and selection process, from the number of records identified in the search to the number of study reports included in the review, ideally using a flow diagram. If applicable, also report the final number of OMIs included and the number<br>of study reports relevant to each OMI. [T]	pp. 12-14
	22b	Cite study reports that might appear to meet the inclusion criteria, but which were excluded, and explain why they were excluded.	p. 14 Supplementary Appendix S1.
OMI characteristics	23a	Present characteristics of each included OMI, with appropriate references. [T]	pp. 14-16
	23b	If applicable, present interpretability aspects for each included OMI. [T]	pp. 16-19
	23c	If applicable, present feasibility aspects for each included OMI. [T]	pp. 20-22
Study characteristics	24	Cite each included study report evaluating one or more measurement properties and present its characteristics. [T]	pp. 22-23

PRISMA - COSMIN OUTCOME Measurement Instruments

Full reports

Section and Topic	#	Checklist item[a]	Location
Risk of bias in studies	25	Present assessments of risk of bias for each included study. [T]	pp. 23-25
Results of individual studies	26	For all measurement properties, present for each study: (a) the reported result and (b) the rating against quality criteria, ideally using structured tables or plots. [T]	pp. 25-27
Results of syntheses	27a	Present results of all syntheses conducted. For each measurement property of an OMI, present: (a) the summarized or pooled result and<br>(b) the overall rating against quality criteria. [T]	pp. 27-31
	27b	If applicable, present results of all investigations of possible causes of inconsistency among study results.	p. 32
	27c	If applicable, present results of all sensitivity analyses conducted to assess the robustness of the synthesized results.	pp. 32-33
Certainty of evidence	28	Present assessments of certainty (or confidence) in the body of evidence for each measurement property of an OMI assessed. [T]	pp. 33-36
Recommendations	29	If appropriate, make recommendations for suitable OMIs for a particular use.	pp. 36-37
DISCUSSION			
Discussion	30a	Provide a general interpretation of the results in the context of other evidence.	pp. 37-38
	30b	Discuss any limitations of the evidence included in the review.	pp.38-39
	30c	Discuss any limitations of the review processes used.	p. 39
	30d	Discuss implications of the results for practice, policy, and future research.	pp. 39- 40

It is strongly recommended that this checklist is used in conjunction with the PRISMA-COSMIN for OMIs 2024 Explanation and Elaboration (E&E) document for important clarification on the checklist items. The PRISMA-COSMIN for OMIs 2024 statement checklist is distributed under the terms of the Creative Commons license.

[a] If an item is marked with [T], a template for data visualization is available. These templates can be downloaded from www.prisma-cosmin.ca.

[b] Item #2.1 in the PRISMA-COSMIN for OMIs 2024 Abstracts checklist refers to the title. Item #2.1 in the Abstracts checklist is identical to item #1 in the Full Report checklist.

From: Elsman EBM, Mokkink LB, Terwee CB, Beaton D, Gagnier JJ, Tricco AC, et al. Guideline for reporting systematic reviews of outcome measurement instruments (OMIs): PRISMA-COSMIN for OMIs 2024. *Quality of Life Research* (2024), doi: https://doi.org/10.1007/s11136-024-03634-y.
