# Supplementary material for: Psychometric Properties of the Breast Cancer Awareness Measure (Breast-CAM): A Systematic Review and Meta-Analysis
Source: Cancers (Basel). 2026 Mar 15;18(6):956. doi: 10.3390/cancers18060956 (PMC13025391; doi:10.3390/cancers18060956)
Supplement: Supplementary file 1 [file cancers-18-00956-s001.zip › Supplementary_File_S2_Search_Strategy.pdf]

Supplementary File 1. Search Strategy:

1.PubMed
Search Date: 2025 sept.15.
Filters applied: Free full text. ***204*** *results*
(((((BCAM[Title/Abstract]) OR (breast cancer awareness measure[Title/Abstract])) OR (breast cancer awareness[Title/Abstract])) OR (Breast CAM[Title/Abstract])breast cancer) AND (("2010"[Date - Publication] : "2025"[Date - Publication]))breast knowledge) AND (("2010"[Date - Publication] : "2025"[Date - Publication]))

2.Embase 377results
Search Date: 2025. Sept.15.
PICO search:
('breast cancer'/exp OR 'female'/exp) AND ('bcam' OR 'breast cancer awareness measure' OR 'breast cam' OR 'validated questionnaire'/mj OR 'questionnaire'/exp OR 'tool'/exp) AND ('breast cancer awareness' OR 'breast cancer knowledge') AND [2010-2025]/py

3.Web Of Science:
Search Date: 2025.Sept.15.
https://www.webofscience.com/wos/woscc/summary/58cc5893-c54c-446c-ba96-f77fdb7b0005-017a3b980b/relevance/1
505 *results from Web of Science Core Collection*
Citation Report: OPEN ACCESS , 2010-2025, ONLY ENGLISH
(((((TI=(breast cancer)) AND TI=(BCAM)) OR TI=(breast cancer awareness measure)) OR TI=(breast CAM)) AND TI=(breast cancer awareness)) OR TI=(breast cancer knowledge)

4.Cochrane Library
Search result: ***37****: 6 Cochrane reviews, 1 Cochrane protocols, 30 Trials,*
Last Saved: 16/09/2025 10:47:32
ID Search
#1 breast cancer:ti,ab,kw AND BCAM:ti,ab,kw OR breast cancer awareness measure:ti,ab,kw AND breast cancer awareness:ti,ab,kw OR breast cancer knowledge:ti,ab,kw with Cochrane Library publication date Between Jan 2010 and Dec 2025 with 'Breast Cancer' in Cochrane Groups

5.Scopus
Result: 32
Search date 16.09.2025
TITLE-ABS-KEY ("breast cancer awareness" W/3 (measure OR tool OR questionnaire)) OR TITLE-ABS-KEY ("Breast CAM") AND TITLE-ABS-KEY ("breast cancer" W/2 (knowledge OR awareness)) AND (SUBJAREA (medi OR nurs OR psyc OR soci)) AND PUBYEAR > 2009 AND PUBYEAR < 2025 AND (LIMIT-TO (LANGUAGE , "English")) AND (LIMIT-TO (OA , "all") OR LIMIT-TO (OA , "publisherfullgold"))

6.EBSCOHOST
Search Date: 2025. 9.16.
Results
144
Search mode
Proximity
Expanders
Apply equivalent subjects
Filters
Peer Reviewed; Full Text; 01/01/2010 - 09/30/2025
Interface
EBSCOhost Research Databases
Databases
Academic Search Complete
TI (TI (breast cancer) AND TX (breast cancer awareness measure) OR TX (breast CAM) OR TX (BCAM) AND TX (breast cancer awareness) OR TX (breast cancer knowledge))

Tools for TI (TI (breast cancer) AND TX (breast cancer awareness measure) OR TX (breast CAM) OR TX (BCAM) AND TX (breast cancer awareness) OR TX (breast cancer knowledge))

2:12 PM
Results: 144
Peer Reviewed
Full Text
01/01/2010 - 09/30/2025
breast cancer
cancer patients
health literacy
questionnaires
health attitudes
women's health
breast cancer patients
public health
health behavior
health education
health promotion
patient education
educational attainment
communication
breast tumor prevention
research evaluation
developing countries
western countries
chinese women
snowball sampling
social aspects
black africans
breast care
china-united states relations
health of african american women
health of patients
english
Proximity
Apply equivalent subjects

7.Clinical Trials.gov
Search Date: 2025.09.17.
*Showing results (46) for: **Breast Cancer | Other terms: Breast Neoplasms | Educational Intervention OR Awareness survey | Female participants | Adult (18 - 64), Older adult (65+) | Study start from 01/01/2010 to 09/30/2025 | Outcome measure: primary OR secondary***
Synonyms of conditions or disease (10)
***breast cancer** ; Breast Neoplasms ; Breast carcinoma ; Breast Neoplasm*
***cancer** ; Neoplasms ; Neoplasm ; Oncology ; Tumors ; Malignant neoplasm ; Tumor ; Cancers*

8.WHO International Clinical Trials Registry Platform (ICTRP)
Search Date: 2025. Oct. 13.
Search Strategy:
Title field: "breast cancer awareness" OR "breast cancer knowledge"
Condition field: "breast cancer"
Intervention field: "breast cancer awareness measure" OR "BCAM"
Recruitment status: Recruiting
No country restrictions
No synonyms excluded
Advanced search interface used
Results Found: 0

9.ISRCTN
Search Date: 2025. Oct. 14.
0 results

Age range: Adult, Condition category: Cancer, Date assigned: from: 01/01/2010, Date assigned: to: 14/10/2025, Interventions: Breast cancer awareness measure OR educational interventions on breast cancer awareness, Outcome measures: Breast cancer knowledge, Participant sex: Female

10.EU-CTR

Search Date: 2025. Oct. 15.
Search result: 0

"breast cancer AND breast cancer awareness measure AND breast cancer education AND breast cancer knowledge AND breast CAM"

11.GOOGLE SCHOOLAR
Search Date: 2025. Sept. 22.
Search result: 101(about)
"breast cancer awareness breast OR cancer OR knowledge OR OR OR breast OR cancer OR awareness OR OR OR breast OR cancer OR screening "breast cancer awareness measure OR breast CAM OR BCAM"

https://scholar.google.com/scholar?start=80&q=breast+cancer+awareness+breast+OR+cancer+OR+knowledge+OR+OR+OR+breast+OR+cancer+OR+awareness+OR+OR+OR+breast+OR+cancer+OR+screening+%22breast+cancer+awareness+measure+OR+breast+CAM+OR+BCAM%22&hl=en&as_sdt=0,5&as_ylo=2010&as_yhi=2024
