# Supplementary material for: Psychometric Properties of the Breast Cancer Awareness Measure (Breast-CAM): A Systematic Review and Meta-Analysis
Source: Cancers (Basel). 2026 Mar 15;18(6):956. doi: 10.3390/cancers18060956 (PMC13025391; doi:10.3390/cancers18060956)
Supplement: Supplementary file 1 [file cancers-18-00956-s001.zip › Supplementary_Table_S1_ Study-level ratings.pdf]

Instrument version	Measurement property	Summarized result	Overall rating	Quality of evidence
**BCAM–UK (Forbes et al., 2011 and related development papers)**	Internal consistency (overall scale)	Cronbach's α reported >0.80 in development/PEP studies; detailed factor structure not consistently reported	? (no consistent structural validity evidence)	Low
**BCAM–UK**	Responsiveness to intervention	Pre–post PEP RCT showed increased awareness scores (older women ≥67 years)	+	Low
**BCAM–UK**	Other properties (structural validity, reliability, measurement error, content validity, criterion validity)	Not tested / not reported	–	Very low
**BCAM–Arabic (2016 Alkhasawneh et al.)**	Content validity	Expert panel, qualitative interviews, cultural adaptation of CAM/BCAM	+	Moderate
**BCAM–Arabic (2016 Alkhasawneh)**	Structural validity	Not performed (no EFA/CFA)	–	Very low
**BCAM–Arabic (2016 Alkhasawneh)**	Internal consistency (warning signs & risk factors)	Cronbach's α = 0.856–0.890	? (no structural validity)	Very low
**BCAM–Arabic (2016 Alkhasawneh)**	Reliability (test–retest)	Inter-rater correlation R = 0.97 (single time point; no repeated measurement)	– (does not meet COSMIN definition)	Very low
**BCAM–Arabic (2016 Alkhasawneh)**	Measurement error	Floor/ceiling effects <15% only; no SEM/SDC	–	Very low
**BCAM–Arabic (2016 Alkhasawneh)**	Hypotheses testing for construct validity	Regression associations with age, education, income (exploratory; no predefined hypotheses)	?	Low
**BCAM–Arabic (2016 Alkhasawneh)**	Criterion validity	Correlation with external criterion r = 0.58, p < .01	+	Moderate
**BCAM–Arabic (2016 Alkhasawneh)**	Responsiveness	Not tested	–	Very low
**BCAM–Arabic (2016 Al-Khasawneh et al.)**	Content validity	Expert panel; forward–back translation; cognitive interviews; behavioural coding; iterative revisions	+	High
**BCAM–Arabic (2016 Al-Khasawneh)**	Structural validity	Not tested	–	Very low
**BCAM–Arabic (2016 Al-Khasawneh)**	Internal consistency – Warning signs	Cronbach's α = 0.890	? (no structural validity)	Very low
**BCAM–Arabic (2016 Al-Khasawneh)**	Internal consistency – Risk factors	Cronbach's α = 0.856	? (no structural validity)	Very low
**BCAM–Arabic (2016 Al-Khasawneh)**	Reliability (test–retest)	Not assessed (reported only inter-rater correlations across groups)	–	Very low
**BCAM–Arabic (2016 Al-Khasawneh)**	Hypotheses testing for construct validity	Correlation awareness ↔ behaviour r = 0.158, p = .001; no predefined hypotheses	?	Low
**BCAM–Arabic (2016 Al-Khasawneh)**	Criterion validity	Correlation with external criterion R = 0.58, p < .01	+	Moderate
**BCAM–Arabic (2016 Al-Khasawneh)**	Other properties (measurement error, responsiveness)	Not tested	–	Very low
**BCAM–Swahili (2017 Wachira et al.)**	Content validity / cross-cultural adaptation	Systematic cognitive testing; language and cultural adjustments	+	High
**BCAM–Swahili**	Structural validity – Symptoms domain	EFA: one factor in both groups (eigenvalues ≈1.5–1.8; loadings 0.65–0.80)	+	Moderate
**BCAM–Swahili**	Structural validity – Barrier's domain	EFA: two factors (internal vs external influences); loadings 0.52–0.84	+	Moderate
**BCAM–Swahili**	Internal consistency – Symptoms	α ≥ 0.80	+	Moderate
**BCAM–Swahili**	Internal consistency – Barriers (external barriers subscale)	α ≈ 0.60	– (below 0.70)	Low
**BCAM–Swahili**	Hypotheses testing for construct validity	Some expected associations supported; not all prespecified; mixed effect sizes	±	Low
**BCAM–Swahili**	Reliability (test–retest), measurement error, responsiveness, criterion validity	Not tested	–	Very low
**BCAM–Persian (2018 Heidari & Feizi)**	Content validity / cross-cultural adaptation	Forward–backward translation; expert review; pilot testing	+	High
**BCAM–Persian**	Structural validity – Warning-signs structure	EFA: two factors ("breast shape changes", "breast pain and lump"); acceptable loadings	+	High
**BCAM–Persian**	Structural validity – CFA	CFA fit: RMSEA = 0.046; CFI = 0.984; TLI = 0.978; good model fit	+	High
**BCAM–Persian**	Internal consistency — Warning signs (overall scale)	Cronbach's α = 0.882	+	High
**BCAM–Persian**	Internal consistency — Shape changes subscale	Cronbach's α = 0.893	+	High
**BCAM–Persian**	Internal consistency — Pain/lump subscale	Cronbach's α = 0.919	+	High
**BCAM–Persian**	Reliability (test–retest, total warning signs)	ICC = 0.841 (95% CI ~0.74–0.91); item κ = 0.47–0.81	+	High
**BCAM–Persian**	Measurement error	SEM = 0.85; SDC = 2.36	± (values reported but no MIC)	Low
**BCAM–Persian**	Hypotheses testing – discriminant validity	ROC AUC = 0.822 (experts vs general women); experts scored higher	+	High
**BCAM–Persian**	Hypotheses testing – convergent validity	All item–scale correlations >0.40	+	High
**BCAM–Persian**	Criterion validity, responsiveness	Not directly assessed	–	Very low
**BCAM–Chinese v1 (2020 Liu et al., early adaptation)**	Content validity	Adopted previously translated C-BCAM; limited description of adaptation	±	Low
**BCAM–Chinese v1**	Structural validity	Limited; no full EFA/CFA; factor structure only briefly described	?	Low
**BCAM–Chinese v1**	Internal consistency — Overall scale	Cronbach's α ≈ 0.91	+	Moderate
**BCAM–Chinese v1**	Internal consistency — Subdomains	Domains α ≈ 0.84–0.94	+	Moderate
**BCAM–Chinese v1**	Hypotheses testing for construct validity	Some associations with expected variables; hypotheses not explicitly prespecified	±	Low
**BCAM–Chinese v1**	Other properties (reliability, measurement error, responsiveness, criterion validity)	Not tested	–	Very low
**BCAM–Chinese v2 (2020 Liu et al., psychometric validation)**	Content validity	Expert panel (≈14 experts, ≥2 rounds); cognitive interviews (n≈15); I-CVI = 0.86–1.00; S-CVI = 0.92	+	High
**BCAM–Chinese v2**	Structural validity – EFA	KMO ≈ 0.78; Bartlett's p < .001; 3-factor solution explaining >50% variance	+	High
**BCAM–Chinese v2**	Structural validity – CFA	χ^2/df ≈1.86; CFI ≈0.94; IFI ≈0.94; RMSEA ≈0.06; SRMR ≈0.05	+	High
**BCAM–Chinese v2**	Internal consistency — Symptoms domain	Cronbach's α = 0.88	+	High
**BCAM–Chinese v2**	Internal consistency — Barriers domain	Cronbach's α = 0.84	+	High
**BCAM–Chinese v2**	Internal consistency — Risk factors domain	Cronbach's α = 0.94	+	High
**BCAM–Chinese v2**	Internal consistency — Overall scale	Cronbach's α = 0.91	+	High
**BCAM–Chinese v2**	Reliability (test–retest)	Test–retest r ≈ 0.72 over appropriate interval (n≈20)	+	Moderate (downgraded for small n)
**BCAM–Chinese v2**	Hypotheses testing for construct validity	No additional predefined hypotheses beyond factor structure	–	Very low

**BCAM–Chinese v2**	Measurement error, responsiveness, criterion validity	Not assessed	–	Very low
**BCAM–Pakistani (2021 Ullah et al.)**	Content validity	Reported content validity index (CVI) ≈ 0.93	+	Moderate
**BCAM–Pakistani**	Structural validity	Not assessed (no EFA/CFA)	–	Very low
**BCAM–Pakistani**	Internal consistency — Overall scale	Cronbach's α ≈ 0.964	? (no structural validity)	Very low
**BCAM–Pakistani**	Hypotheses testing for construct validity	Associations between BCAM scores and demographic variables; exploratory	?	Low
**BCAM–Pakistani**	Other properties (reliability, measurement error, responsiveness, criterion validity)	Not assessed	–	Very low
**BCAM–Turkish (2021 Baş & Ursavaş)**	Content validity	Expert panel; I-CVI = 1.00 for all items; S-CVI = 1.00	+	High
**BCAM–Turkish**	Structural validity – CFA	11-item one-factor model; χ^2/df ≈ 4.72; RMSEA = 0.078; CFI/IFI/NFI/NNFI ≈ 0.90; all loadings significant	+	High
**BCAM–Turkish**	Internal consistency — Overall scale	Cronbach's α = 0.89	+	High
**BCAM–Turkish**	Reliability (test–retest)	ICC = 0.89; item κ = 0.55–0.96	+	High
**BCAM–Turkish**	Hypotheses testing for construct validity	Known-groups validity (health professionals' higher awareness than general women)	+	Moderate
**BCAM–Turkish**	Measurement error, responsiveness, criterion validity	Not assessed	–	Very low
**BCAM–Malay (BCAM-M; 2022 Yusuf et al.)**	Content validity / cross-cultural adaptation	Adapted BCAM-M; forward translation, cultural adaptation, pilot testing (n=10)	+	Moderate
**BCAM–Malay**	Structural validity	Not tested (no EFA/CFA)	–	Very low
**BCAM–Malay**	Internal consistency — Overall scale	Cronbach's α = 0.89	? (no structural validity)	Very low
**BCAM–Malay**	Responsiveness to intervention	Pre–post educational intervention: significant improvements in BCAM-M scores	+	Low
**BCAM–Malay**	Other properties (reliability, measurement error, construct validity, criterion validity)	Not assessed	–	Very low
**BCAM–Sudanese (2023 Lawis & Sabir)**	Content validity / cross-cultural adaptation	Translation + expert review + pilot testing	+	Low
**BCAM–Sudanese**	Structural validity	Not tested (no factor analysis)	–	Very low
**BCAM–Sudanese**	Internal consistency — Overall scale	Cronbach's α = 0.825	? (no structural validity)	Very low
**BCAM–Sudanese**	Hypotheses testing for construct validity	Associations with age, education and contact; exploratory, no predefined hypotheses	?	Very low
**BCAM–Sudanese**	Other properties (reliability, measurement error, responsiveness, criterion validity)	Not assessed	–	Very low
**BCAM–Greek (2024 Papasozomenou et al.)**	Content validity / cross-cultural adaptation	Forward–back translation; pilot testing in rural Greek setting	+	Moderate
**BCAM–Greek**	Structural validity	Not tested (no EFA/CFA)	–	Very low
**BCAM–Greek**	Internal consistency — Warning signs	Cronbach's α = 0.89	? (no structural validity)	Very low
**BCAM–Greek**	Internal consistency — Risk factors	Cronbach's α = 0.85	? (no structural validity)	Very low
**BCAM–Greek**	Hypotheses testing for construct validity	χ^2 associations between BCAM responses and demographic variables; exploratory only	?	Very low
**BCAM–Greek**	Other properties (reliability, measurement error, responsiveness, criterion validity)	Not assessed	–	Very low

Notes: *Results are synthesized at the level of instrument versions rather than individual publications. Subscale-level evidence is reported for multidimensional versions (e.g., Persian, Chinese, Greek). "–" indicates the measurement property was not assessed in the relevant study. COSMIN ratings:* ***(+) sufficient***, ***(–) insufficient***, ***(±) inconsistent***, ***(?) indeterminate****. Quality of evidence was graded per measurement property based on risk of bias, inconsistency, indirectness, and imprecision using a PROM-adapted GRADE approach.* ***Abbreviations:*** *α = Cronbach's alpha; ICC = Intraclass correlation coefficient; κ = Cohen's kappa; SEM = Standard error of measurement; SDC = Smallest detectable change; AUC = Area under the ROC curve; EFA = Exploratory factor analysis; CFA = Confirmatory factor analysis; CVI = Content validity index; I-CVI = Item-level CVI; S-CVI = Scale-level CVI; RMSEA = Root mean square error of approximation; CFI = Comparative fit index; IFI = Incremental fit index; NFI = Normed fit index; NNFI/TLI = Non-normed (Tucker–Lewis) fit index; SRMR = Standardized root mean square residual; BCAM = Breast Cancer Awareness Measure; PEP = Promoting Early Presentation intervention.*
